# Targeting tumor-associated macrophages to reverse antitumor drug resistance

**DOI:** 10.18632/aging.205858

**Published:** 2024-05-23

**Authors:** Sheng Li, Jiyao Sheng, Dan Zhang, Hanjiao Qin

**Affiliations:** 1 The Second Hospital of Jilin University, Changchun, China; 2 Department of Hepatobiliary and Pancreatic Surgery, Second Hospital of Jilin University, Changchun, China; 3 Department of Radiotherapy, The Second Hospital of Jilin University, Changchun, China

**Keywords:** tumor-associated macrophages, tumor microenvironment, immunotherapy, drug resistance, cancer

## Abstract

Currently, antitumor drugs show limited clinical outcomes, mainly due to adaptive resistance. Clinical evidence has highlighted the importance of the tumor microenvironment (TME) and tumor-associated macrophages (TAMs) in tumor response to conventional antitumor drugs. Preclinical studies show that TAMs following antitumor agent can be reprogrammed to an immunosuppressive phenotype and proangiogenic activities through different mechanisms, mediating drug resistance and poor prognosis. Potential extrinsic inhibitors targeting TAMs repolarize to an M1-like phenotype or downregulate proangiogenic function, enhancing therapeutic efficacy of anti-tumor therapy. Moreover, pharmacological modulation of macrophages that restore the immune stimulatory characteristics is useful to reshaping the tumor microenvironment, thus further limiting tumor growth. This review aims to introduce macrophage response in tumor therapy and provide a potential therapeutic combination strategy of TAM-targeting immunomodulation with conventional antitumor drugs.

## INTRODUCTION

Chemotherapy, immunotherapy, and targeted therapy are currently widely applied in the clinical treatment of cancer. However, a large number of clinical trials have demonstrated that the long-term application of these drugs makes it difficult to avoid resistance. Moreover, the treatment effect of a single drug application is poor, with few lasting treatment responses and survival benefits. A large number of clinical studies have demonstrated that the development of drug resistance is closely associated with the tumor microenvironment (TME) and tumor-associated macrophages (TAMs).

The TME comprises an extracellular matrix (ECM), stromal cells, and immune cells (including T and B lymphocytes, natural killer cells, and TAMs) [1]. With the gradual advancement of research on the TME in recent years, its vital role in the development and evolution of tumors is becoming increasingly important. TAMs are important components of the TME. Two main sources are involved: one is derived from monocytes in the blood recruited to the tumor site under the effect of chemokines, while the other is derived from tissue-resident macrophages inherent to the corresponding tumor site, which originate during embryonic development. Macrophages demonstrate remarkable plasticity and exhibit two extreme phenotypes when exposed to various stimuli, which are broadly classified as pro-inflammatory M1 (classically activated macrophages) and anti-inflammatory M2 (alternatively activated macrophages) [2]. Inflammation in tumors leads to cell composition change in the TME. Hypoxia and lactic acid accumulation in TME change the TAM populations and phenotype. Increased TAMs offer a good environment for the invasion and metastasis of tumors [3]. Therefore, nearly all drug resistance is closely related to the complex interaction of the TME and TAMs.

This study investigates the interaction between the TME and TAMs and the role of TAMs in tumor progression. We also reviewed the clinical evidence of reprogramming and drug resistance induced by TAMs during tumor treatment. We summarized the recent studies as well as the mechanism of TAM reprogramming and induced drug resistance during treatment. Finally, we discussed the combination of drugs targeting TAM and traditional antitumor drug therapy as an emerging cancer treatment strategy.

## TME and TAMs

In the TME, inflammation leads to changes to TAMs, including the recruitment from monocytes in the blood and the proliferation of tissue-resident macrophages. This cancer-related inflammation always promotes the development and progression of tumors. In acute inflammation, the expression of pro-inflammatory cytokines and chemokines increases, including IL-1, IL-6, C-C motif ligand 2 (CCL2), CCL5, C-X-C motif chemokine ligand 8 (CXCL8), CD40, and TNFα. These cytokines or chemokines recruit monocytic macrophages into tumors and have a crucial role in the TME [4, 5]. In addition to the chemotaxis and recruitment of monocytes in blood circulation, tissue-resident macrophages have an important role in the formation of the TME [6]. Evidence has shown that IL-4, a cytokine secreted by Th2 cells, drives the proliferation and expansion of tissue-resident macrophages in inflammation led by Th2 cells [7]. The tissue-resident macrophages are among the first cells to interact with transformed cells in tumors, and they may play a vital role in tumorigenesis [6]. Different from acute inflammation, chronic inflammation has a long-term effect on TME formation and is always closely associated with tumors in chronic inflammation. Chronic inflammation is a major risk factor for colorectal cancer. The p38αMAPK/MAKAP Kinse2 (MK2) axis is an important component of cell stress. In response to the stimulatory signals of chronic inflammation, it promotes the polarization of TAMs to pro-angiogenic M2-like macrophages in colorectal cancer and then stimulates the progression of chronic inflammation to colorectal cancer [8]. In breast cancer, obesity causes the CCL2 signal to initiate chronic inflammation, thus leading to the recruitment to the TME of many macrophages and fibroblasts, thus resulting in collagen deposition and tumor fibrosis in the TME and significantly promoting tumor progression [9]. Chronic inflammation also causes an increase in reactive oxygen, resulting in DNA damage, cell death, and loss of repair programs. This reduces the antitumor ability of cytotoxic T cells and promotes immune escape of tumors [4]. In conclusion, these studies suggest that acute and chronic inflammation in the TME could impact the components of TAMs through numerous mechanisms, and TAMs promote tumor growth, invasion, angiogenesis, metastasis and immunosuppression through several mechanisms [10].

In mice, tissue-resident macrophages in the lung interstitium have been shown to offer nutritional support to tumor cells and promote tumor growth. Additionally, macrophages recruited by CCL2 primarily promote tumor invasion and spread in the lungs [10]. In breast tumors, macrophages are attracted by colony-stimulating factor-1 (CSF-1) released by cancer cells. The released epidermal growth factor (EGF), in response, stimulates cancer proliferation. This paracrine cycle between macrophages and cancer cells is mediated by EGF, and CSF-1 promotes tumor invasion [11, 12]. In terms of angiogenesis promotion, TAMs primarily expressing angiopoietin receptor Tie2 have been reported to accumulate in the perivascular region of tumors and promote tumor angiogenesis. This function can be further upregulated by exposure to tumor-derived factors such as angiopoietin-2 (ANGPT2) [13, 14]. TAMs also have an important part in promoting distant tumor dissemination and metastasis. The experimental results demonstrate that circulating tumor cells reside in the area with rich tissue-resident macrophages. These cells may thus contribute to the establishment of the pre-metastatic niche by maintaining an optimal “soil” for the eventual seeding of disseminated tumor cells [6]. Along with tissue-resident macrophages, TAMs, which primarily express Tie2, promote the increase in vascular permeability and the infiltration of tumor cells via vascular endothelial growth factor A (VEGF-A) signaling and then promote the distant spread of tumor cells [15]. Along with the above-mentioned mechanisms, TAMs mediate immune suppression in tumor treatment, help tumors to escape from immune response, and have a therapeutic effect. Both recruited macrophages and tissue-resident macrophages express inhibitory checkpoint molecules, namely programmed cell-death-ligand 1 (PD-L1), PD-L2, and PD-1, which inhibit the antitumor activity of T cells and affect immune checkpoint blockade (ICB) therapy. In conclusion, macrophages in the TME can impact all aspects of tumor growth and invasion through several mechanisms, and the TME is a complex system, the characteristics of which affect macrophages.

The accumulation of a large amount of lactic acid caused by hypoxia is the primary cause of TAM differentiation into the pro-tumor phenotype. Hypoxia in the TME is primarily classified into two types, namely chronic hypoxia and cycling hypoxia [16]. Chronic hypoxia is caused by the large distance between cells and blood vessels, the growth of which cannot cope with the rapid growth of tumors, resulting in a continuous decline in oxygen levels [16], while circulatory hypoxia is a local intermittent hypoxia caused by an abnormal vascular structure [17]. Hypoxia can promote the further recruitment of TAMs and enable the function of TAMs to be more oriented towards the M2 phenotype, thus promoting tumor progression. Chronic hypoxia increases the expression of CXCL4 in uveal melanoma cells [18], CXCL9 and CXCL10 in liver cancer cells [19, 20], and CXCL1 and CXCL2 in TAMs [21]. Circulating hypoxia increases the expression of CXCL8 in TAMs [21]. The majority of these chemokines of the CXC family have the function of promoting TAM recruitment to the TME [16]. Chronic hypoxia is continuously accompanied by the activation of the NF-κB signaling pathway, which improves the response of TAMs to the above cytokines and further promotes their recruitment [22]. Additionally, a study on hypoxia in lung cancer reports that hypoxia reduces the secretion of proinflammatory factors of M1-like TAMs and promotes the polarization to M2-like TAMs [23]. Along with the effect of hypoxia on TAMs, hypoxia could produce a large amount of lactic acid through anaerobic glycolysis to promote tumor progression. Hypoxia inducible factor-1α (HIF-1α) is an essential transcription factor for vascular endothelial growth factor (VEGF), and lactic acid can stabilize HIF-1α, make it enter the nucleus, and induce TAMs to secrete VEGF [24]. HIF-1α entering the nucleus under the effect of lactic acid induces TAMs to express arginase, which can catalyze the conversion of arginine to ornithine. Ornithine is conducive to the production of polyamines, which have a crucial role in cell proliferation and are conducive to tumor growth and invasion [24]. Additionally, experiments have indicated that TAMs express higher levels of M2-like genes Arg1 and Mrc1 under the influence of lactic acid, which promotes TAMs to differentiate into M2-like phenotypes [24]. Therefore, lactic acid is among the “signals” for communication between tumors and macrophages. Macrophages monitor tumors via lactic acid and “maintain homeostasis” to preserve tumor growth [24].

## Clinical evidence for TAMs in tumor response to therapies

Changes to the TME have an impact on the number and subtype of TAMs. In the clinical treatment of tumors, the majority of drugs change the TME and then change the number and phenotype of macrophages via recruitment and polarization. These recruited and tissue-resident macrophages contribute to tumor growth and metastasis, thus affecting the prognosis of tumor patients. In several recent clinical trials of tumor therapy, TAMs have been the focus of researchers. In several studies, macrophages were used as markers to indicate the therapeutic impact of tumors or the prognosis of patients. Evidence of macrophage recruitment and polarization can be found in clinical trials of multiple mainstream cancer therapies (including chemotherapy, immunotherapy, and vascular targeted therapy) (Table 1).

**Table 1:** Clinical evidence for the role of TAMs in tumor responses to therapies.

**Treatment**	**Drug**	**Tumor**	**TAM Reeducation**	**Significance**	**References**
**Chemotherapy**	Doxorubicin, bleomycin, vinblastine	Classical Hodgkin’s lymphoma	CD68+ macrophages are significantly increased	The increase in CD68+ macrophages is associated with poor prognosis and could be a single prognostic biomarker	David W. Scott 2013
	Durvalumab, nab-paclitaxel	Breast cancer	Compared with pCR, the cancers with RD had higher expression of macrophage markers such as CCL-3, CCL-5, CXCL-1, CXCL-6, IL-1, IL-34 and more TAM infiltration	Increased TAMs and upregulated expression of TAM-related genes are associated with poor prognosis	Kim R. M. Blenman 2022
	Cabazitaxel	Gastroesophageal cancer	M2-like TAMs are enriched in tumors	The enrichment of macrophages suggests that the tumor is responsive to treatment	Manish A. Shah 2020
	NAC	Invasive breast cancer	Increased TAMs and CD68+ phenotype in the tumor bulk and infiltrative areas and increased TAMs after neoadjuvant chemotherapy (NAC) is associated with response to NAC in invasive breast cancer	TAMs and CD68 could be used as an immune predictive signature for response to NAC in invasive breast cancer	Lauren E. McLemore 2018
	Chemotherapy and rituximab	Diffuse large B-cell lymphoma	CD68+ macrophages are increased significantly after chemotherapy	The prognosis of patients with increased CD68+ macrophages after chemotherapy was poor, while the prognosis of patients with increased CD68+ macrophages after immunotherapy was good	Sari Riihijärvi 2015
**Immunotherapy**	Anti-PD-1 blockade	Intrahepatic cholangiocarcinoma	The number of TAMs increased, and the expression of SPP1 gene was upregulated. The MIF signaling pathway between tumor cells and TAMs or T cells is increased	Upregulation of SPP1 gene and increase in MIF signaling pathway promote tumor progression	Bao-Ye Sun 2022
	Keytruda, cyclophosphamide	Sarcoma	The infiltration of CD163+ macrophages increases after treatment. Meanwhile, the expression of indoleamine 2, 3-dioxygenase 1 (IDO1) in macrophages increases	Indoleamine 2, 3-dioxygenase 1 (IDO1) expressed by macrophages can produce kynurenine. Activation of the IDO1/kynurenine pathway may be an important mechanism leading to resistance to anti-PD-1 therapy	Maud Toulmonde 2018
	Atezolizumab, carboplatin, paclitaxel	Non-small-cell carcinoma (NSCLC)	Both the number and volume of CAMLs increase	Increased numbers of CAMLs are associated with tumor progression and poor survival	Alexander Augustyn 2021
	Toripalimab, carboplatin, pemetrexed	Non-small-cell carcinoma (NSCLC)	The infiltration of M2-like macrophages decreases, and M1/M2 ratio increases significantly	The reduction in M2-like macrophage infiltration is associated with longer survival and better prognosis	Tao Jiang 2021
	Camrelizumab, radiation and chemotherapy	Esophageal carcinoma	The proportion of PD-L1-negative macrophages in tumor is increased after treatment	Patients with a higher proportion of PD-L1-negative macrophages have a longer survival time, and the closer tumor cells are to PD-L1-negative macrophages, the better the prognosis	Xiaoxue Ma 2022
	Bevacizumab and ipilimumab	Metastatic melanoma	Extensive macrophage infiltration after treatment	The infiltration of macrophages affects the prognosis of patients	F Stephen Hodi 2014
	Anti-CD19 chimeric antigen receptor T cells (CAR-T)	B-cell non-Hodgkin's lymphoma	TAM infiltration increases after treatment, and CD68 + and CD163 + cells increased more significantly in the PR group than in the CR group	Increased TAM infiltration is negatively correlated with remission status, which can affect the efficacy of CAR-T	Zi-Xun Yan 2019
**Vascular targeted therapy**	Bevacizumab	Rectal cancer	Upregulated gene expression of SDF1α and its receptor CXCR4 and CXCL6 in cancer cells, and induced NRP1 expression in TAMs	VEGF blockade upregulates inflammatory pathways and NRP1	Xu 2009
		Recurrent glioblastoma	Increased TAMs and CD163+ phenotype in the tumor bulk and infiltrative areas, and increased TAMs after antiangiogenic therapy is associated with poor survival	TAMs participate in immune escape from antiangiogenic therapy and represent potential biomarkers of resistance	Lu-Emersom 2013
		Glioblastoma	Accumulation of Tie2+ macrophages in surgical glioblastoma specimens and overexpressed MMP9	TEMs are associated with glioma recurrence after anti-VEGF therapy	Gabrusiewicz 2014
		Glioblastoma	Downregulated macrophage MIF and an increase in harbored M2 protumoral macrophages	Resistance is driven by reduced MIF causing proliferative expansion of M2 macrophages	Castro 2017
		Metastatic colorectal cancer	Genes that regulate TAMs-related functions are significantly associated with clinical outcomes	TAM-related gene variations predict outcomes of bevacizumab treatment	Sunakawa 2015
	Gefitinib/Erlotinib	Advanced non-small-cell lung cancer	Most TAMs were located in the tumor stroma and positively costained with the M2 marker CD163, and TAM counts were significantly higher in patients with progressive disease	TAMs are correlated with response to EGFR-TKI and independently predict survival in patients treated with EGFR-TKI	Chung 2012
		Advanced lung adenocarcinoma	M2 were significantly higher in patients with progressive disease, and M2 were shown to be significantly related to poor progression-free survival and overall survival	M2-TAMs are related to response of EGFR-TKIs and independently predict survival in patients treated with EGFR-TKI	Zhang 2014
		EGFR mutated lung adenocarcinoma	Blood monocytic S100A9+ MDSC counts were higher and were associated with poor treatment response and shorter progression-free survival	TAMs and S100A9+ MDSC-mediated EGFR-TKI resistance	Feng 2018
	Cetuximab	Colorectal cancer	M2 macrophages revealed high levels of Fc-gamma receptors (FcgRs) and PD-L1 and production of IL-10 and VEGF but not IL-12	Tumor-promoting M2 macrophages are activated by the therapeutic mAb cetuximab in the local tumor microenvironment	Pander 2011

### Chemotherapy

Chemotherapy drugs are currently the most broadly applied drugs in clinical cancer treatment. Even though targeted therapy and immunotherapy have emerged in the past few years, they still cannot replace the salient position of chemotherapy drugs. Recent clinical trials have demonstrated changes in the number of TAMs or M1/M2 phenotypes in tumors post-chemotherapy. These changes are always suggestive of tumor response to chemotherapy drugs, and some are closely associated with poor prognosis of tumor patients. A clinical trial of a chemotherapy regimen for advanced classical Hodgkin’s lymphoma demonstrated that an increase in CD68+ macrophages in the TME post-treatment was closely associated with poor prognosis, and CD68 could even be applied as a poor prognostic marker [25]. Along with chemotherapy alone, TAMs are equally important in the combination of chemotherapy and immunotherapy. In a study of durvalumab combined with paclitaxel, doxorubicin, cyclophosphamide, as well as other chemotherapeutic agents in triple-negative breast cancer, RNA and DNA sequencing technology indicated that patients with residual disease (RD) exhibited upregulated CCL-3 and CCL-5 compared with patients with complete response (CR). This promotes the recruitment and infiltration of macrophages to the tumor site and indicates that the increased number of macrophages is related to the poor prognosis of patients [26]. Along with the poor prognostic significance of TAMs in chemotherapy, several studies have shown that an increase in TAMs may represent the impact of the drug in treatment. In a clinical trial on cabazitaxel in the treatment of gastroesophageal cancer, the enrichment of M2-like macrophages in tumors suggested a response to treatment [27]. In breast cancer, TAMs and their marker CD68 were increased in tumors treated with taxane and carboplatin, which can be used as markers of response to chemotherapy [28]. Notably, in a study on the combination of chemotherapy and targeted therapy in diffuse large B-cell lymphoma (DLBCL), an increase in CD68+TAMs post-chemotherapy alone indicated a poor prognosis, while an increase in CD68+TAMs content post-rituximab treatment had a positive effect on the prognosis of patients [29].

### Immunotherapy

The changes to TAMs after immunotherapy were also of great significance for the efficacy and prognosis of patients, and the changes in various subtypes of TAMs have different levels of significance for prognosis. Immune checkpoint blockade (ICB) has been the representative of immunotherapy in the past few years. A clinical study assessed the changes to cells in the TME post-ICB treatment of intrahepatic cholangiocarcinoma by single-cell RNA sequencing technology. The number of TAMs increased, and the expression of SPP1 gene was upregulated and has a core role in tumor progression. Additionally, the MIF signaling pathway between tumor cells and TAMs or T cells was increased, which has been shown to promote cancer progression [30]. A clinical trial of the PD-1 blocker pembrolizumab in combination with cyclophosphamide chemotherapy for sarcoma reported an increased infiltration of CD163-positive macrophages and immunosuppressive effects in treated tumors and that these macrophages expressed indoleamine 2 and 3-dioxygenase 1 (IDO1) to produce kynurenine. The activation of the IDO1/kynurenine pathway may be an important mechanism leading to resistance and anti-PD-1 therapy [31]. Additionally, a type of cancer-associated macrophage-like cells (CAMLs), which are circulating multinucleated myeloid cells, can be detected in the blood of some patients with malignant tumors. A study on atezolizumab and carboplatin or paclitaxel in combination with non-small-cell lung cancer demonstrated an increase in the number and size of CAMLs. They are also associated with tumor progression and a poor survival rate [32]. A study on a toripalimab (PD-1 blocker), carboplatin, and pemetrexed combination in the treatment of non-small-cell lung cancer reported that M2-like macrophage infiltration was reduced, M1/M2 ratio was significantly increased, and the survival time of patients was prolonged after immunotherapy was combined with chemotherapy [33]. In a clinical trial on camrelizumab combined with chemoradiotherapy in the treatment of esophageal cancer, the proportion of PD-L1-negative macrophages in the tumor lumen increased post-treatment, and the survival time was prolonged. Additionally, the closer the tumor cells were to PD-L1-negative macrophages, the better the prognosis [34]. Along with PD-1 blocker therapy, TAMs changes appeared in the clinical trials of several other types of immunotherapies. For example, the biopsy results of CTLA4 inhibitor ipilimumab combined with bevacizumab in the treatment of metastatic melanoma demonstrated extensive TAM infiltration [35]. In a clinical trial on anti-CD19 chimeric antigen receptor T cells (CAR-T) for B-cell non-Hodgkin’s lymphoma, RNA sequencing demonstrated an increase in TAM infiltration post-treatment, with a greater increase in CD68 + and CD163 + cells (the latter representing M2-like TAMs) in the PR group compared with the CR group. This indicates that increased TAM infiltration is negatively correlated with remission status, and this impacts the efficacy of CAR-T [36].

### Vascular targeted therapy

Recombinant humanized monoclonal antibodies and VEGFR/EGFR tyrosine kinase inhibitors (TKIs) have been widely applied in the treatment of metastatic colorectal cancer, glioblastoma, as well as non-small-cell lung cancer [37]. Although the list of vascular-targeted drugs is still expanding, latent adverse effects have been reported in clinical practice. Numerous studies have demonstrated a significantly higher number of CD68+ macrophages and CD163+ M2-like phenotypes in the tumor mass and within infiltrative areas of glioblastoma patient post-bevacizumab therapy [38–41]. Microarray analysis indicated that a reduced level of macrophage migration inhibitory factor can be the most pertinent mediator of this, thus resulting in polarization to protumoral M2 and specific localization to the glioblastoma rim [38]. The accumulation of highly proangiogenic Tie2-expressing macrophages (TEMs) was demonstrated in malignant glioma surgical samples that relapsed post-anti-VEGF treatment, and the TEMs were shown to secrete more gelatinase enzymatic proteins to improve the invasive characteristics of glioma cells [39]. After bevacizumab treatment of rectal cancer patients, a high level of circulating stromal cell-derived factor 1α (SDF1α) was reported in association with rapid distant disease progression. This was predicted, since the SDF1α pathway is a known independent regulator of angiogenesis and metastasis [42]. Bevacizumab also induced significant changes to neuropilin 1 (NRP1) expression in TAMs, and NRP1 was shown to modulate the alternatively activated M2 phenotype of macrophages. Such clinical evidence supports the role for TAMs in mediating the tumor response to vascular targeted therapy.

The therapeutic outcome of anti-angiogenic agents has been modest and transitory, with drug resistance reported to develop after a period of pharmaceutical response [43]. Numerous studies in the literature have suggested that tumor-promoting TAMs are involved in angiogenesis and immune suppression, inducing resistance to vascular-targeted therapy [38, 42–46]. When mediating immune escape from anti-VEGF therapy, TAMs may apply additional mechanisms rather than depend on the VEGF pathway to rescue tumor invasion, migration, and angiogenesis [47–49]. Intravital imaging recorded tumor cells interacting with TAMs to exit their primary site into blood vessels and seed to distant metastatic organs, a process relying on the function of macrophages [14, 50]. The DSC-MR imaging technique offers direct evidence that CCL2 expression by TAMs can raise the resistance to bevacizumab [51]. The genetic deletion of macrophages or medical inhibition of their recruitment has strongly attenuated these seeding events and primary tumor growth after being exposed to vascular targeted therapy [52–54].

In cancer patients treated with anti-vascular therapy, clinical evidence revealed that TAMs were significantly associated with poor clinical prognosis, including progression-free survival and overall survival in a broad range of tumors, such as advanced lung adenocarcinoma, colorectal cancer, and glioblastoma [40, 44, 55–57]. TRIBE and FIRE3 trials also demonstrated variations in genes regulating TAM-related functions that could predict the clinical outcomes of bevacizumab-based treatment in patients with metastatic colorectal cancer [56]. Moreover, a proangiogenic subset of macrophages was found to be closely related to glioma recurrence after anti-VEGF therapy, attributable to their direct role in tumor invasion and aggression [39, 40].

### EGFR-TKIs

Epidermal growth factor receptor–tyrosine kinase inhibitors (EGFR-TKIs) are the most common drugs applied in targeted therapy. The EGFR-TKI representative drugs gefitinib and erlotinib increased the TAM count in the tumor stroma of patients with EGFR-mutated lung cancer and significantly attenuated therapeutic cytotoxicity [44, 55, 57]. In response to EGFR-TKIs, M2-TAMs, but not M1-TAMs, were higher in patients diagnosed with progressive disease than in those without it [55, 57]. In a phase III randomized clinical trial on metastatic colorectal cancer, the anti-EGFR monoclonal antibody cetuximab activated tumor-promoting M2 macrophages in the local TME, thus releasing multiple anti-inflammatory factors and pro-tumor mediators, and the infiltration of M2 macrophages was closely associated with poor prognosis [58, 59]. Additionally, a clinical trial on sunitinib or panitinib in patients with renal cell carcinoma (RCC) reported increased TAMs in tumor samples post-treatment, and it was shown to be associated with poor prognosis [60, 61].

## Mechanisms by which therapies reprogram TAMs

Numerous clinical trials have demonstrated that the number of macrophages in tumor sites increases, and the phenotype changes post-conventional treatment, which are often closely associated with the prognosis of patients. Researchers have discussed the mechanism of the above phenomenon via clinical research. The research primarily concentrated on two aspects of chemotherapy and vascular targeted therapy (Table 2). Chemotherapy impacts the expression of specific genes in tumor cells and macrophages in the TME, regulates the secretion of cytokines, promotes the recruitment of monocytes in the blood to the tumor site, and polarizes the infiltrating macrophages to the M2 phenotype. The primary function of vascular-targeted therapy is the reduction in the tumor vascular bed, while subsequent hypoxia can be significantly increased [62–65]. Hypoxia also modulates innate immune cells to induce angiogenesis and immune evasion, and this regulates the production of cytokines and growth factors by tumor cells and further reprograms TAMs via paracrine activation [66–68]. Hypoxia can exert selective pressure on cancer cells with only aggressive phenotypes able to survive, thus causing invasive properties and limiting the efficacy of anti-cancer therapy [64, 65, 69]. Subsequently, we discuss the mechanisms by which chemotherapies and vascular targeted therapies reprogram TAMs in preclinical studies and concentrate on how to avoid the reeducation of TAMs.

**Table 2:** TAM reprogramming driven by tumor therapies.

**TAM Reprogramming**	**Drug**	**Tumor**	**Effects on tumor**	**Potential extrinsic inhibitors**	**References**
**Increased TAM recruitment**	Gemcitabine	Pancreatic cancer	After treatment with gemcitabine, pancreatic cancer cells promoted the recruitment of macrophages and tumor progression through IL-8	IL-8 neutralizing antibody	Sachin Kumar Deshmukh 2018
	Neoadjuvant chemotherapy	Breast cancer	The recruitment of monocytes increased through the expression of YKL-39. TAMs promote the angiogenesis and metastasis of breast cancer	YKL-39 blocker	Liu 2018
	Paclitaxel, pexidartinib	Solid tumor	The expression of CSF-1 increased, leading to the recruitment of TAMs and drug resistance, while combined treatment with pexidartinib could inhibit the recruitment	Pexidartinib	Robert Wesolowski 2019
	Paclitaxel, cisplatin	Lewis lung cancer	TAMs increased after treatment with paclitaxel and cisplatin, while the number of macrophages does not change in the heparanase knockout mice	Heparanase inhibitor	Udayan Bhattacharya 2020
	Sorafenib	Metastatic liver cancer	Tumor progression	Clodrolip or ZA	Zhang 2010
		Liver cancer	Tumor growth, neovascularization, progression, and drug resistance	Anti-Ly6G antibody	Zhou 2016
	Bevacizumab	Breast cancer	Tumor malignancy and drug resistance	Blockade of Oncostatin M/Eotaxin	Tripathi 2014
	Sunitinib	Glioblastoma	Tumors developed aggressive mesenchymal features and increased stem cell		Piao 2012
**Polarization to M2-TAMs**	Cisplatin, carboplatin	Cervical and ovarian cancer	Cervical and ovarian cancer cell lines showed increased production of PGE2 and IL-6, which promote macrophages to polarize towards the M2 subtype and promote drug resistance	COX inhibitors, anti-IL-6R	Eveline M Dijkgraaf 2013
	Paclitaxel	Breast cancer	A large accumulation of ROS in the TME. ROS stimulate the secretion of cytokines such as IL-10, IL-4, IGFBP-3, and CXCL1	Anti–PD-L1 blockade	Cecilia Roux 2019
	Neoadjuvant chemotherapy	Esophageal squamous cell carcinoma	APOE, APOC1, and SPP1 are highly expressed in TAMs, leading to the increase in M2 macrophages and drug resistance		Chen 2021
	Temozolomide	Melanoma	Exocytic vesicles shed by melanoma cells after temozolomide treatment promote macrophages towards the M2 phenotype, leading to tumor repopulation and treatment failure		Luciana Nogueira de Sousa Andrade 2019
	Bevacizumab	Glioblastoma	Induces resistance to antiangiogenic therapy	AZD1480	de Groot 2012
		Breast cancer	Tumor malignancy and drug resistance	Blockade of Oncostatin M/Eotaxin	Tripathi 2014
	Sorafenib	Pancreatic neuroendocrine tumor	Immune suppression, reneovascularization, and tumor regrowth	IPI145	Rivera 2015
**Promotion of proangiogenic TAM subset**	CA4P	Breast cancer	Limited therapeutic efficacy of agent	AMD-3100	Welford 2011
	Bevacizumab	Glioblastoma	Enhanced invasive properties and secretion of high levels of gelatinase enzymatic proteins		Gabrusiewicz 2014
	DC101	Glioblastoma	Increased signal for tumor-remodeling properties and invasive tumor growth	Inhibitor of Ang-Tie2 axis	Cortes-Santiago 2016
**Enhanced proangiogenic TAM activities**	Clone 3.19.3	Breast cancer	Upregulated expression of several proangiogenic genes, such as Vegfa, Vegfb, Pdgfb, Sdf1, and Mmp9		Mazzieri 2011
	Bevacizumab	Ovarian cancer	Enhanced expression of alternative proangiogenic chemokines and cytokines including G-CSF and PDGF	ZA (depletion of macrophages)	Daltion 2017

### Increased TAM recruitment

Monocytic lineage cells trafficking from the circulation into the tumor stroma are under the control of tumor-derived chemoattractants, and preclinical evidence revealed the impact of chemotherapy and anti-angiogenic drugs on proangiogenic macrophage recruitment.

Chemotherapy leads to the recruitment and infiltration of macrophages primarily by promoting the upregulation of several chemokines. In an experiment on pancreatic cancer mice treated with gemcitabine, the expression of IL-8 in pancreatic cancer cells and the infiltration of macrophages at the tumor site were increased. Meanwhile, the infiltration of macrophages was reduced after the treatment with an IL-8 neutralizing antibody. This indicates that pancreatic cancer cells completed the recruitment of macrophages by upregulating the expression of IL-8 [70]. After the treatment of breast cancer mouse models with neoadjuvant chemotherapy drugs, macrophages expressed more YKL-39, which is a newly discovered chemokine promoting the recruitment and infiltration of monocytes from blood to tumor sites [71]. In a paclitaxel-treated mouse model of breast cancer, tumor cells upregulated the expression of colony-stimulating factor 1 (CSF-1) and resulted in the recruitment of TAMs and resistance to paclitaxel. Conversely, the introduction of the CSF-1R inhibitor pydatinib in combination with paclitaxel blocked macrophage recruitment and significantly increased CD8+ T cells. This demonstrates that CSF-1 has a crucial role in the recruitment of macrophages induced by chemotherapy [72]. Another experiment on the treatment of lung cancer mice by paclitaxel combined with cisplatin demonstrated that the macrophages in tumors significantly increased post-treatment, while the number of macrophages in heparanase knockout mice did not change post-treatment, suggesting that the activation of macrophages by chemotherapy was dependent on heparanase [73]. Additionally, a study on durvalumab (PD-L1 blocker) combined with albumin paclitaxel and doxorubicin/cyclophosphamide in the treatment of triple-negative breast cancer also demonstrated that the expression of CCL3, CCL5, and other chemokines increased post-chemotherapy, and these chemokines promoted the recruitment of macrophages to the tumor site [26].

Multi-target VEGFR therapy has been demonstrated to rapidly induce intratumor hypoxia and improve the infiltration of macrophages into tumors, which may be vital to the development of drug refractoriness and consequent tumor progression [54, 74–76]. The remaining tumor cells can exploit hypoxia to their own advantage [64]. In the hypoxic milieu, tumor cells produce and secrete multiple chemokines and cytokines to entrap macrophages from the bloodstream into the tumor [69]. Sorafenib was reported to significantly increase the peripheral recruitment and intratumoral infiltration of macrophages in metastatic liver cancer, accompanied by an elevation of SDF-1α, CSF-1, and VEGF in the tumor stroma [54]. In the hypoxic niches induced by sorafenib, TAMs promote the expression of galectin-3, and this further induces TAM infiltration [77]. Furthermore, the TAM-mediated promotion of tumor growth and metastasis in hypoxia was inhibited by the administration of a macrophage-depletion agent or galectin-3 inhibitor in a mammary model [77]. The depletion of macrophages by zoledronic acid and clodrolip in combination with sorafenib may significantly suppress tumor angiogenesis and distant organ metastasis, demonstrating the role of TAMs in tumor development in sorafenib therapy [54]. Sorafenib also mediated the infiltration of tumor-associated neutrophils (TANs) in hepatocellular carcinoma patients and animal models, while TANs further promoted more intratumoral macrophages and T-regulatory (Treg) cells through secreting CCL2 and CCL17 [76]. The injection of neutralizing antibodies against CCL12 and CCL17 into tumors caused an explicit reduction in tumor size and pulmonary metastasis, with a drop in the migratory activities of TAMs and Treg cells [76]. Additionally, sunitinib and bevacizumab treatments simultaneously inhibited the infiltration of macrophages and enhanced overall survival compared with bevacizumab therapy alone. However, gliomas developed a mesenchymal phenotype and expressed stem cell markers at the late tumor of tumor progression [74].

### Polarization of TAMs to M2-like phenotype

Macrophages show remarkable plasticity in response to microenvironmental cues [78]. An alteration in macrophage polarization in the TME to an immune-suppressive phenotype was always reported after chemotherapy or vascular targeted therapy [75, 79, 80].

Unlike chemotherapy-induced recruitment of TAMs that is primarily dependent on various chemokines, chemotherapy-induced polarization of TAMs to the M2 phenotype has more diverse mechanisms. The experimental results suggest that prostaglandin E2 (PGE2) and IL-6 increased in cervical and ovarian cancer cell lines treated with carboplatin or cisplatin. These two inflammatory mediators promoted the polarization of macrophages to M2 subtype, while blocking the typical NF-κB signaling pathway could inhibit the impact of chemotherapy and prevent the production of PGE2 and IL-6 in tumor cell lines. Meanwhile, the enhancement of M2 phenotypic polarization by chemotherapy disappeared. These phenomena suggest that chemotherapy depends on two inflammatory mediators, PGE2 and IL-6, to promote M2-type polarization [81]. Additionally, paclitaxel treatment of breast cancer mice causes a large accumulation of reactive oxygen species **(**ROS) at the tumor site, which stimulates the secretion of cytokines such as IL-10, IL-4, insulin-like growth factor binding protein 3 (IGFBP-3), and CXCL1 and promotes the transformation of macrophages to the immunosuppressive phenotype [82]. Global single-cell transcriptome analysis using data from clinical trials demonstrated that TAMs highly expressed APOE, APOC1, and SPP1 genes in patients with esophageal squamous cell carcinoma (ESCC) post-neoadjuvant chemotherapy, and this leads to an increase in M2-like macrophages [83]. Additionally, in an experiment applying temozolomide to treat melanoma mice, it was found that exocytic vesicles exfoliated by melanoma cells post-treatment polarize macrophages towards the M2 phenotype by upregulating M2-like genes such as ARG-1 and IL-10 [84].

The mechanism of TAM polarization to M2 type induced by vascular-targeted drugs is similar to that of macrophage recruitment. Local hypoxia caused by vascular blockade remains the most important mechanism. A recent study suggested that hypoxia-primed breast cancer cells chemoattracted and polarized macrophages to a proangiogenic M2-like phenotype by elevating cytokines eotaxin and oncostatin M [75]. The blockade of eotaxin and oncostatin M augmented the therapeutic efficacy of anti-angiogenic bevacizumab for supporting tumor regression [75]. The hypoxic microenvironment has a vital role in cancer stemness acquisition and epithelial–mesenchymal transition, thus promoting tumor progression [85]. In glioblastomas after bevacizumab therapy, hypoxia can persistently induce the expression of phosphorylated signal transducer and activator of transcription 3 (p-STAT3) in glioma cells, which preserves the mesenchymal transformation of normal neuroglial cells and the stemness of glioma cells [79]. STAT3 was also responsible for the modification of TAMs to the tumor supportive M2-like subtype, while the administration of the STAT3 inhibitor combined with anti-angiogenic agents explicitly decreased microvascular density and tumor proliferation, with a lower number of p-STAT3 macrophages [79]. A recent study based on the hepatocellular carcinoma model indicated that sorafenib increased the synthesis and secretion of hepatocyte growth factor (HGF) by TAMs. HGF activates phosphoinositide-3-kinase/protein kinase B (PI3K/AKT) pathways in tumor cells and promoted the polarization of TAMs to the M2-like phenotype [86]. In a pancreatic neuroendocrine cancer model, sorafenib promoted the polarization of TAMs, TANs, as well as other myeloid cells to an immune-stimulating phenotype. However, this effective response was transient in that all these myeloid cell populations were skewed to the immune-suppressive type, causing tumor regrowth and neo-vascular formation [80]. The pharmacological inhibition of phosphoinositide-3-kinase in myeloid cells can improve the endurance and efficacy of vascular-targeted therapy by reversing the subset of these myeloid cells [80].

### Promoting proangiogenic TAM subset

TEMs are a proangiogenic subpopulation of macrophages characterized by both expression of macrophage markers and the angiopoietin receptor Tie2 and are found in the circulation and the tumor stroma of both humans and mice [87, 88]. Prior studies have suggested that clusters of TEMs surrounding blood vessels make contact with nearby endothelial cells to sustain the tumor microvessel network, which was correlated with therapeutic intolerance and poor prognosis [50, 89, 90]. The upregulation of proangiogenic TEMs is a mechanism by which vascular-targeted therapies reshape macrophages. The leading vascular disrupting agent (VDA), combretastatin A4 phosphate (CA4P), is a microtubule depolymerizing drug that causes the cytoskeletal and morphological destruction of vascular structures [91–93]. After treatment with CA4P in murine breast cancer models, tumors significantly increased their levels of chemokine CXCL12 and repopulated the infiltration of proangiogenic TEMs [94]. Inhibiting TEM recruitment by genetically depleting TEMs or pharmacologically inhibiting the CXCL12/CXCR4 axis significantly assisted the vascular-destroying impact of CA4P [94]. Recent research reported that hypoxia-inducible transcription factors HIF-1α in hypoxic conditions promote the infiltration of proangiogenic TEMs, while the HIF-2α suppresses this particular mechanism [95]. The two different types of HIFα subunits regulate Tie2-expressing macrophages [95]. The anti-VEGF therapy bevacizumab induced the invasive outgrowth of malignant gliomas via TEM accumulation [39]. In this pro-invasive TME, Ang2 was explicitly increased post-anti-VEGF treatment [96] and chemoattracted TEMs into tumors and aggravated signals of tumor-remodeling properties [97]. This suggests a vital role for the Ang2–Tie2 pathway in recurrent tumors following anti-angiogenesis therapies and offers rational therapeutic strategies.

### The regulation of TAM proangiogenic activities

Prior work has suggested that TAMs facilitate resistance to anti-VEGF therapy by altering proangiogenic activities [98, 99]. The macrophage level increased with the emergence of VEGFA-targeted monoclonal antibody refractoriness in an ovarian-cancer-bearing mice model. The resistant macrophages involved in the development of refractoriness indicated heightened viability, aggression, and migration [100]. Compared with sensitive TAMs, resistant TAMs demonstrated a significantly enhanced expression of alternative proangiogenic chemokines and cytokines, including granulocyte CSF and platelet-derived growth factor (PDGF), rather than the secretion of VEGFR [100]. Moreover, the blockade of Ang2 upregulated the expression of several pro-vascular genes in a breast cancer mice model, such as Vegfa, Vegfb, Pdgfb, Sdf1, and Mmp9 [101]. Reported preclinical evidence suggests that inflammatory cytokines derived from monocytes or macrophages promoted vascular CXCR4 expression on endothelial cells, while reducing CXCR4 expression by the depletion of macrophages enhanced the anti-tumor impact of sorafenib in liver cancer [102].

## Mechanisms of TAM-mediated drug resistance

During tumor treatment, drugs reprogram TAMs, and the mechanism of reprogrammed TAMs mediating drug resistance of tumor cells becomes more complex. In the following section, we discuss how TAMs mediate drug resistance of tumor cells using three treatment methods, namely chemotherapy, immunotherapy, and vascular targeted therapy.

### Chemotherapy

The TAM mechanisms mediating chemoresistance comprise regulating tumor cell apoptosis, participating in epithelial–mesenchymal transition (EMT), metabolites competing with chemoresistance, and regulating autophagy, among others. Some researchers investigated the mechanism of TAMs mediating the resistance of colorectal cancer cells to 5-FU. Metabolomics analysis demonstrated that putrescine, one of the metabolites of TAMs, increased post-5-FU treatment. Further animal experiments demonstrated that putrescine could reduce the activation of apoptotic protein caspase-3, thus protecting tumor cells from the impact of the apoptosis mechanism. Some researchers investigated the mechanism of TAMs mediating the resistance of colorectal cancer cells to 5-FU. Metabolomics analysis suggests that putrescine, one of the metabolites of TAMs, increased post-5-FU treatment. Further animal experiments demonstrated that putrescine inhibits the activation of apoptotic protein caspase-3, thus protecting tumor cells from the impact of the apoptosis mechanism. The inhibition of putrescine production promotes the apoptosis of tumor cells, i.e., it restores the sensitivity of colorectal cancer cells to 5-FU. These studies demonstrated that TAMs inhibit the apoptosis of tumor cells through putrescine, one of its metabolites, and then causes colorectal cancer cells to become resistant to 5-FU [103]. One study reported that the cytokine CCL22 secreted by M2-type macrophages inhibited the cleavage of three apoptotic proteins, caspase-3, caspase-8, and PARP, and increased the expression of p-PI3K and p-AKT, thus inhibiting 5-FU-induced apoptosis of colorectal cancer cells and mediating drug resistance [104]. Additionally, this study indicated that macrophages mediate 5-FU resistance in colorectal cancer by participating in epithelial–mesenchymal transition (EMT) [104]. In a study on gemcitabine in pancreatic cancer treatment, researchers applied C13 isotope labeling of glucose carbon atoms. They reported that compared with the M1-like macrophages, M2-like macrophages are more marked in the metabolism of glucose into the tricarboxylic acid cycle, and these marked carbon atoms were applied to synthesize more pyrimidine. Deoxycytidine (deoxycytosine nucleoside) was similar to gemcitabine in molecular structure and inhibits gemcitabine activity via molecular competition, thus leading to resistance to gemcitabine [105, 106]. In an oxaliplatin resistance experiment of liver cancer cells, after the co-culture of TAMs and hepatocellular carcinoma (HCC) cells, it was reported that HCC autophagy was activated, HCC was resistant to oxaliplatin, and the sensitivity of HCC cells to oxaliplatin was significantly enhanced when ATG5 siRNA was applied to inhibit autophagy of HCC cells. With this, TAMs can mediate oxaliplatin resistance in HCC cells by inducing autophagy [107].

### Immunotherapy

In immunotherapy, the impact of TAMs on the prognosis of patients in various types of tumors is different. In a study on the mechanism of glioblastoma resistance, the combination of PD-1 and CTLA-4 antibodies was applied to treat glioblastoma mice. TAMs from the resistant mice were isolated and co-cultured with T cells, and it was reported that CD80 on the surface of T cells increased. CD80 was previously shown to substitute for PD-1 in binding to its ligand PD-L1, thus inhibiting CD4+T cell proliferation accompanied by an increase in Treg cells. Subsequently, PD-L1 antibody therapy was introduced to the previous regimen, which inhibited the interaction between CD80 and PD-1. The inhibition of CD4+T cell proliferation and the increase in Treg cell expansion were weakened, and the impact of ICB therapy was further enhanced. These studies reported that macrophages induced the inhibition of CD4+T cells and the expansion of Treg cells in the TME via the PD-L1/PD-1/CD80 signaling pathway, thus mediating the resistance of glioblastoma to the combination therapy of PD-1 and CTLA-4 blockades [108]. Additionally, in a clinical trial, the puncture biopsy of patients with hepatocellular carcinoma treated with PD1 blockade demonstrated that the expression of circTMEM181 (a circular RNA) was significantly upregulated in some drug-resistant individuals. A series of animal and cell experiments demonstrated that circTMEM181 is an exosome secreted by HCC cells. It can absorb Mir-488-3p (a miRNA) in macrophages, thus upregulating the expression of CD39 in macrophages, a key enzyme in initiating the ATP-adenosine pathway. CD39 attenuates extracellular ATP (eATP), stimulating immune response signals in the TME by hydrolyzing eATP and ADP to AMP. The immune responses are weakened, thus mediating the resistance of HCC cells to PD-1 blockade therapy [109]. In an experiment in which PD-1 antibody and CTLA-4 antibody were combined to treat breast cancer mice, the expression of IFNγ was significantly upregulated post-treatment. IFNγ induces macrophages to produce more CXCL9 and CXCL10, two chemokines that bind to CXCR3 on the surface of T cells and promote their recruitment for anti-tumor immune response. The application of IFNγ neutralizing antibodies, knockdown of CXCL9/CXCL10 gene, or CXCR3 blockade of macrophages all cause the attenuation of anti-tumor immune response. This indicates that the production of CXCL9 and CXCL10 by macrophages induces T cell recruitment and is an important mechanism of immune checkpoint blockade in breast cancer treatment [110].

### Vascular targeted therapy

TAM-mediated drug resistance in vascular targeted therapy is primarily related to the inhibition of tumor cell apoptosis. The treatment of lung adenocarcinoma with the EGFR inhibitor erlotinib cannot avoid drug resistance and gradually leads to tumor progression. In view of the causes of its drug resistance, some researchers explored its mechanism: TAMs in the TME secretes EREG (one of the ligands of EGFR), induces ERbB2 to form EGFR/ERbB2 heterodimer, downregulates cleaved caspase nine and other apoptotic proteins through its downstream PI3K/AKT pathway, inhibits the apoptosis of tumor cells, and then mediates the resistance of NSCLC to EGFR-TKI inhibitors [111]. Another study on EGFR-TKI inhibitors in NSCLC reported that their resistance was closely associated with alveolar macrophages (AMs) in the TME. In the EGFR signaling pathway, AM produced local proliferation and was gradually polarized into tumor-promoting the M2 phenotype, which mediates drug resistance by inhibiting tumor cell apoptosis and other mechanisms [112]. An animal experiment of bevacizumab, a representative drug of vascular targeted therapy, in the treatment of triple-negative breast cancer, demonstrated that it induced the co-activation of TLR4 and Fcγ receptors on the surface of macrophages, which made macrophages polarized to the M2b subtype (CD11b+ CD86+ IL10 high). This subtype of macrophages produces great amounts of TNFα, which induces the expression of immunosuppressive molecule IDO1. It has been shown to promote drug resistance and is associated with poor prognosis. The inhibition of TNFα decreases the expression of IDO1 and enhances the therapeutic impact of bevacizumab. These findings show that M2b macrophages can induce bevacizumab resistance in breast cancer cells via the TNFα-IDO1 axis [113]. Additionally, researchers have investigated the resistance mechanism of bevacizumab in the treatment of glioblastoma (GBM) and found that GBM cells secrete two cytokines, IL-8 and CCL2, which stimulate TAMs to produce TNFα, which activates endothelial cells (ECs). EC activation has been reported to be associated with bevacizumab resistance and poor prognosis. This indicates that TNFα secretion by TAMs and activation of ECs is a salient molecular mechanism of bevacizumab resistance in GBMs [114].

## Therapeutic potential of targeting TAMs

**Figure 1:**
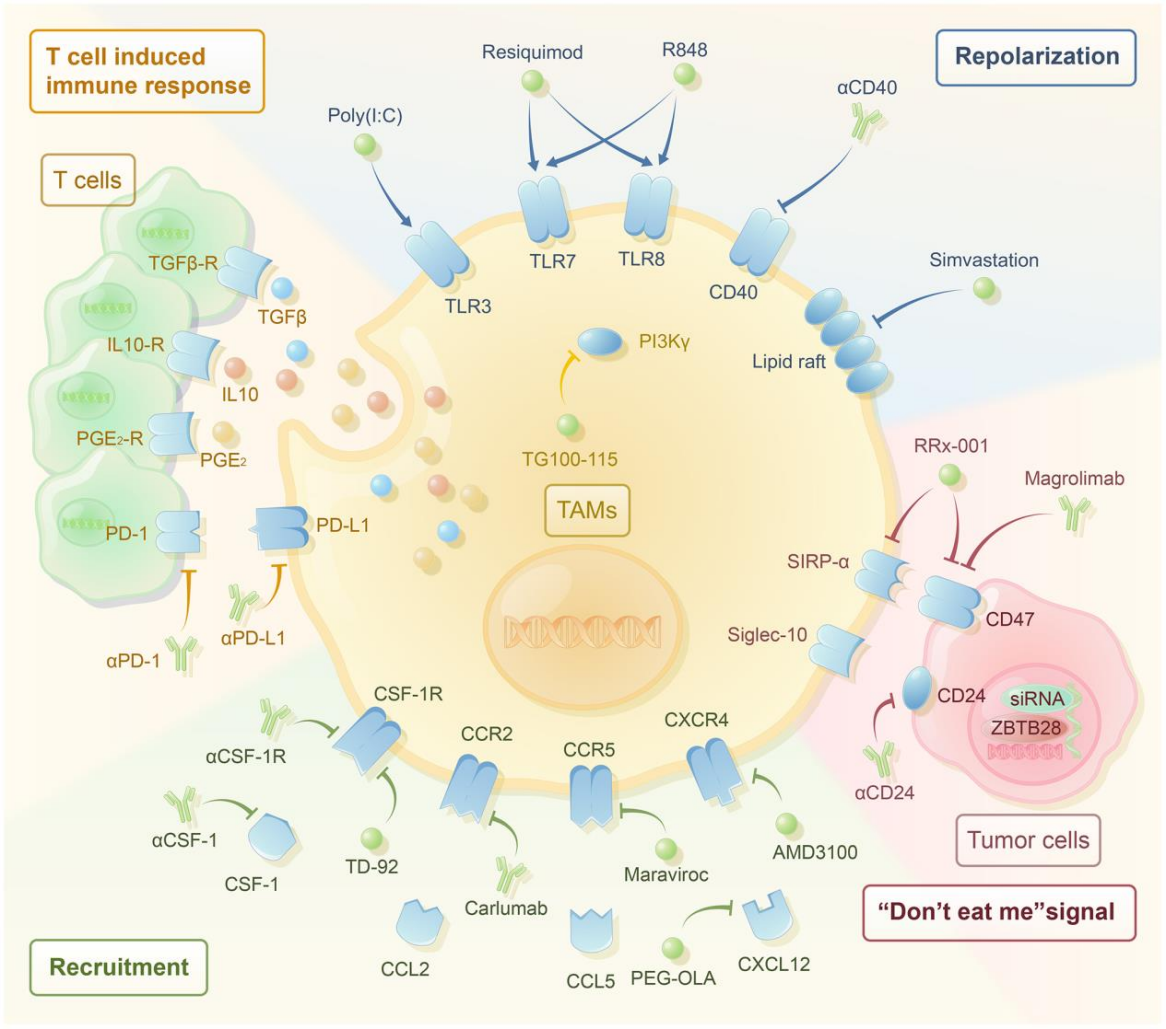
**The therapeutic strategies targeting macrophages have been noticeable in recent years.** Single application and combined application with traditional drug therapy have great potential. According to their mechanisms, they are mainly divided into the following categories. Reducing the recruitment of TAMs includes the use of αCSF-1, αCSF-1R, TD-92 (CSF-1R inhibitor), carlumab (αCCR2), maraviroc (CCR5 inhibitor), OLA-PEG (CXCL12 inhibitor), and AMD3100 (CXCR4 inhibitor). Reprogramming TAMs to the M2-like phenotype includes the use of TG100-115 (PI3Kγ inhibitor), poly (I:C) (TLR3 agonist), resiquimod (TLR7/8 agonist), R848 (TLR7/8 agonist), αCD40, and simvastation. TAMs inhibit the T-cell-induced immune response through PD-1/PD-L1, PGE2, IL-10, and TGF-β. The use of αPD-1, αPD-L1, and macrophage targeted therapy may activate T-cell-induced immune response. Blocking the “don’t eat me” signal includes the use of αCD24, magrolimab (αCD47), Rx-001 (both SIRP-α and CD47 inhibitor), and siRNAs against JMJD1A.

Through investigating the mechanism of TAM reprogramming and resistance induced by TAMs, researchers have produced many new drugs targeting TAMs for tumor treatment. These drugs regulate the immune function of TAMs and induce their transformation in an anti-tumor direction. They are currently primarily applied in combination with chemotherapy, immunotherapy, vascular targeted therapy, and other conventional treatment methods to overcome the resistance of conventional treatment methods and have an adjuvant therapeutic effect. According to the various mechanisms and targets, macrophages can be classified into reducing or depleting TAMs, repolarizing TAMs towards M1-like macrophages, enhancing the immune function of T cells, and other potential therapeutic targets (Figure 1). Additionally, the application of nanotechnology and other biomaterials can help achieve the delivery of drugs targeting TAMs to the tumor site and monitor the immune function of TAMs, which is another important strategy to enhance anti-tumor therapy development.

### Reducing the recruitment of TAMs

The primary approach to reduce the number of TAM infiltrates is to inhibit the recruitment of monocytes. Several preclinical studies demonstrated that chemokine inhibitors reduce TAM infiltration and effectively inhibit tumor progression [41, 115, 116]. The combination of TAM chemokine inhibitors with chemotherapy or immunotherapy has significant potential in tumor treatment. CSF-1, also known as M-CSF, is a key proliferation and differentiation factor for monocyte–macrophage lineages [117, 118] that recruits macrophages to the tumor site and promotes TAM polarization. Therefore, CSF-1/CSF-1R represents a target for the direct or indirect medical intervention of TAMs [78]. A recent preclinical study of CSF-1 inhibition combined with immunotherapy in non-small-cell lung cancer reported that TD-92 (a novel erlotinib derivative) enhanced the antitumor effect of PD-1 blocker by downregulating CSF-1R and depleting TAMs in the TME [119]. Other animal experiments demonstrated that the introduction of CSF-1R inhibitors after anti-VEGF drug resistance reduced the tumor size to little or no measurable degree in high-grade serous ovarian cancer [120]. To simulate clinically adaptive conditions, mice were treated with bevacizumab and paclitaxel until the emergence of resistance, then CSF-1R inhibitors markedly attenuated the function of macrophages and restored the response to anti-angiogenesis treatment, thus causing robust anti-tumor effects [120]. In addition to CSF-1, both CCL2 and CCL5, as two important chemokine ligands, have an important role in TAM recruitment. Many scholars have investigated them as targets to inhibit TAMs, but most of their impacts were negative [121–123]. Additionally, blocking the chemokine-receptor axis CXCR4/CXCL12 inhibited the recruitment of TAMs. For example, introducing an SDF-1α (CXCL-12) inhibitor to anti-VEGF antibodies significantly reduced the level of TAMs and prolonged the survival time of glioblastoma-bearing rodents compared with the VEGF blockade alone [116]. Inhibiting CXCR4-dependent immunosuppressive monocytes similarly enhanced anti-angiogenic therapy in colorectal cancer by regulating the recruitment of these myeloid cells [124].

### Reprogramming TAMs to M1-like phenotype

Chemokines can activate phosphoinositol 3-kinase γ subtype (PI3Kγ) in myeloid cells within the TME, thus promoting the recruitment of monocytes to tumor sites, and are closely related to the M2-type polarization of TAMs [125]. In the past few years, the inhibition of PI3Kγ to assist other drugs in tumor treatment has gained widespread attention. An animal experiment has proven that the combination of PI3Kγ inhibitor and temozolomide in the treatment of glioblastoma overcame the resistance of temozolomide and enhanced the anti-tumor effect [126]. Poly (l-glutamic acid)-combretastatin A4 conjugate (PLG-CA4), as a novel class of vascular disrupting agents (VDAs), can induce the polarization of TAMs towards the M2-like phenotype in breast cancer. Compared to the monotherapy of PLG-CA4, the combination of PLG-CA4 and PI3Kγ inhibitor potentially enhances the impact of cytotoxic T lymphocytes and significantly extends the mean survival time through decreasing the number of M2-like TAMs [127]. TLRs are important pathogen recognition receptors expressed by immune system cells. The stimulations to TLR3, TLR4, TLR7/8, and TLR9 lead to the rapid activation of innate and adaptive immunity. A recent study observed that some M1-type markers (CD86, CD80, and CD40) were upregulated in macrophages in response to TLR3 stimulation both *in vivo* and *in vitro*, while M2-type specific indicators such as CD206 decreased [128]. Fibrosarcoma mice treated with a combination of TLR3 agonist poly (I:C) and TLR7/8 agonist resiquimod demonstrated increased M1 macrophages, decreased M2 macrophages, and increased infiltration of CD8+T cells and CD4+T cells. Compared with single TLR agonist treatment, the impact of the above combined treatment was more significant, and the anti-tumor effect was significantly better [129]. Several studies have demonstrated that oxaliplatin resistance is primarily related to the reduction in myeloid derived suppressor cells (MDSCs) and their differentiation into M1-type macrophages. Treatment with TLR7/8 agonist combined with oxaliplatin can significantly promote the differentiation of MDSCs into M1-type macrophages, thus overcoming drug resistance [130]. Additionally, researchers have used nanoscale metal–organic frameworks (nMOFs) to co-deliver TLR7 and CD47 antibodies to the tumor site and cooperated with PD-L1 immune checkpoint inhibitors to treat colorectal cancer. This repolarized M2-like macrophages into the M1 type, achieving excellent anti-tumor efficacy [131]. The cell surface molecule CD40 is a highly conserved costimulatory protein on antigen presenting cells. Recent studies have reported that agonistic CD40 antibodies can activate macrophages and promote the repolarization of TAMs to M1 type, thus enhancing anti-tumor immune responses [132–134]. In preclinical studies, the combination of CD40 agonist antibodies and imatinib in gastrointestinal stromal tumor treatment promoted TAMs towards the M1 type, significantly overcoming resistance to tyrosine kinase inhibitors individually [135]. Poly (ADP-ribose) polymerase inhibitor (PARPi) is approved for the treatment of ovarian or breast cancers with BRCA1/2 mutations (BRCAmut), many studies have demonstrated that PARPi olaparib could cause reprogramming of TAMs to higher cytotoxicity and phagocytosis with anti-tumor efficacy [136]. Researchers have found olaparib induce increased glycolysis and oxidative phosphorylation, leading to more reactive oxygen species (ROS) and TAMs transcriptional reprogramming. Further analysis has revealed that administration of CD47 antibodies combined with olaparib may improve tumor control [137]. In addition to the typical targets above, one preclinical study suggested the application of the cholesterol-lowering drug simvastatin to reduce cholesterol synthesis [138]. On the one hand, it inhibits the lipid raft/integrin/FAK pathway; on the other hand, it inhibits the cholesterol-dependent LXR/ABCA1 mechanism (hepatic X receptor/ATP-binding cassette transporter A1), repolarizing TAMs, thus reducing the generation of TGFβ. The two mechanisms jointly overcome the paclitaxel resistance induced by epithelial–mesenchymal transition (EMT). Paclitaxel combined with simvastatin in non-small-cell lung cancer treatment achieved good efficacy.

### Targets of activating T-cell-induced immune response

Macrophages, as innate immune cells, present antigens to stimulate the activation signal of T cells and are potent effector cells that activate adaptive immune responses [139–141]. However, the infiltration of M2-like TAMs results in an immunosuppressive TME that inhibits the activation of cytotoxic T lymphocytes during tumor treatment [142–144]. This can be achieved through expression of inhibitory immune checkpoint molecules, including PD-L1 [145] and secretion of immunosuppressive cytokines (including IL-10, TGF-β, prostaglandin E2) [146]. Through the therapeutic targeting of anti-inflammatory TAMs, the disinhibition of CD8+ T-cell-dependent immune responses may enhance the therapeutic efficacy of vascular-targeted therapies. The CCL2/CCR2 axis mediates TAM infiltration, while the application of a natural CCR2 antagonist decreased the number of TAMs in the tumor stroma and shifted them towards the M1-like phenotype, further elevating the number of CD8+ T cells [147]. The CCR2 antagonist, combined with low-dose sorafenib, potentiated anti-liver tumor effects via upregulating intratumoral CD8+ T cells and enhanced the distribution of CD8+ T cells in the tumor milieu, without obvious toxicity [147]. C-C motif chemokine receptor-like 2 (CCRL2) is a nonsignaling atypical receptor cloned from LPS-activated macrophages. Clinical data indicate that melanoma patients with high CCRL2 expression exhibit increased infiltration of CD8+T cells with stronger antitumor activity. Researchers have proven that CCRL2 retains TLR4 on the surface of the macrophage membrane via animal experiments and then activates macrophages to the M1 phenotype via the Myd88-NFκB signaling pathways. It amplifies the antitumor response of CD8 + T cells, and CCRL2 may thus be a potential target for cancer immunotherapy [148]. A recent preclinical study on the CSF-1/CSF-1R inhibitor pexidartinib in the treatment of sarcoma demonstrated that, in addition to decreasing TAM infiltration and phenotypic changes from M2 to M1, increased CD8+T cell infiltration and decreased Treg cell infiltration were observed in the TME post-treatment. These results may offer a reference for antitumor therapy targeting macrophages to enhance CD8+T cell activity [149]. CXCL12/CXCR4 is a key signaling pathway that recruits TAMs, and the blockade of the CXCL12/CXCR4 possesses great potential for cancer treatment [150]. A recent preclinical study on hepatocellular carcinoma demonstrated that a novel CXCR4 antagonist combined with sorafenib increased cytotoxic CD8+ T cell infiltration more significantly, and the overall survival was more remarkably extended than using sorafenib alone [150]. Such a combination based on the relief of TAM-mediated immunosuppression enhanced the anti-tumor function of vascular-targeted therapy. Currently, the blockade of immune checkpoint signals has demonstrated durable therapeutic responses in clinics [151]. Although these immunotherapies have demonstrated great potential to treat tumors, immune checkpoint inhibitors only function when CD8+ T cell are infiltrated within the TME [152]. Numerous studies have shown that the repolarization of macrophages to the M1 phenotype restores the infiltration and cytotoxicity of CD8+ T cells, thus limiting tumor progression and metastasis [151–153]. Adding checkpoint inhibitors to the combination between vascular-targeted agents and vascular-targeted therapy may yield improved clinical benefits. A preclinical study investigated the application of CSF-1R inhibitors PLX3397 (pexidartinib) and PLX5622 in combination with anti-PD-1 immunotherapy in lung squamous cell carcinoma. The inhibition of CSF-1R reduced the infiltration of macrophages at the tumor site, inhibited the activity of CD8+T cells by M2 macrophages, and enhanced the migration and infiltration of CD8+T cells into tumor islets. Its combination with anti-PD-1 therapies further increased the close contact between CD8+T and tumor cells, eventually delaying tumor progression [154].

### Other potential molecular targets

CD24 and CD47 are two types of membrane proteins broadly expressed by tumor cells, which can bind to signal proteins on the surface of macrophages, trigger the “don’t eat me” signal, and inhibit macrophage phagocytosis, thus escaping anti-tumor immunity. Therefore, the inhibition of CD24 and CD47 has become a new strategy of anti-tumor immunotherapies and has prompted great research potential in recent years. Preclinical studies have demonstrated that CD24 promotes immune evasion in ovarian cancer and triple-negative breast cancer by interacting with the inhibitory receptor sialic acid binding ig-like lectin 10 (Siglec-10) expressed by tumor-associated macrophages (TAMs). Both genetic ablation of CD24 or Siglec-10 and the monoclonal antibody blockade of the CD24–Siglec-10 interaction enhance the phagocytosis of macrophages to tumor cells, inhibit tumor growth, and prolong overall survival time [155]. A research team developed a CD47 mAb using hybridoma technology and tested it in preclinical studies. It was combined with a standard chemotherapy regimen to treat mice with triple-negative breast cancer and significantly inhibited tumor growth [156]. Another preclinical study demonstrated that the combination of CD47 mAb Hu5F9-G4 and trastuzumab in the treatment of Her2+ breast cancer cells significantly overcame the resistance to trastuzumab monotherapy and effectively inhibited tumor growth [157]. CD24 inhibits the antitumor effect of macrophages by binding to signal-regulatory protein alpha (SIRPα) on the surface of macrophages. Transcription factor ZBTB28 is a tumor suppressor with extensive expression in normal tissue. A series of preclinical studies have demonstrated that the gene expression of CD24 and CD47 is beneficial to increase the anti-tumor phagocytosis of macrophages. However, the gene is always silent in breast cancer, so activating this gene can have a significant anti-tumor effect. This study provides novel ideas and research directions for the immunotherapy of breast cancer [158]. In addition to the two targets above, researchers have reported several unique targets for macrophage therapy in the field of vascular targeted therapy, which are mechanistically suitable for the combined application with vascular targeted therapy. Following the management of vascular-targeted therapies, hypoxia and nutrient starvation collaborate to prompt aggressive properties that resist anti-angiogenic attacks [159, 160]. Recent research has thus focused on alleviating hypoxia outcomes or even normalizing the tumor vasculature to improve anti-angiogenic therapy. Microarray analysis identified *in vitro* upregulation of the functional driver JMJD1A, a histone demethylase, under long-term hypoxic conditions and *in vivo* prior to the activation of angiogenesis or the refractory phase of anti-angiogenic drugs [161]. JMJD1A inhibition explicitly suppressed tumor progression by decreasing macrophage infiltration and the angiogenic switch, thus enhancing the anti-tumor effects of anti-VEGF agents [161]. The medical inhibition of the VEGF/VEGFR pathway failed to prolong overall survival in patients with glioblastoma [162, 163], and studies demonstrated that circulating Ang-2 levels rebounded after the administration of the pan-VEGFR inhibitor cediranib [164]. Moreover, combination therapy using cediranib and an anti-Ang-2 neutralizing antibody morphologically and structurally transformed and normalized the highly aberrant and dysfunctional tumor vessel network [165]. Dual targeting of Ang-2 and the VEGF pathway reshaped the pro-tumor M2 subtype towards anti-tumor M1 macrophages, thus reducing the tumor burden and mediating survival benefits in glioblastoma [165, 166].

### The clinical trials of TAM-targeting therapy

Clinical trials have recently been completed for a variety of drugs targeting TAMs (Table 3). The targets of TAMs mainly include CSF-1R, CCL, CD40, TLR, PI3Kγ, and CD47, most of them are in phase I clinical trials with good therapeutic efficacy. Among the drugs that inhibit the recruitment of TAMs, the CSF-1R inhibitor pexidartinib in clinical trials has excellent clinical benefits in solid tumors such as in tenosynovial giant-cell tumors (TGCT), unresectable sarcoma, and malignant peripheral nerve sheath tumors. The current study has found that pexidartinib combined with paclitaxel result in a completed remission (CR) rate of 3% and a partial remission (PR) of 13%. Especially, the most promising signal of clinical activity has been noted in patients with platinum-resistant or -refractory epithelial gynecologic malignancies, which patients have experienced a CR. Moreover, the phase III trial of pexidartinib for the treatment of TGCT has been completed [167–169]. The clinical trial has shown that Pexidartinib is shown a robust tumour response in TGCT with improved patient symptoms and functional outcomes and could be considered as a potential treatment. Emactuzumab (CSF-1R inhibitor) has also shown excellent efficacy against TGCT [170] but has shown poor efficacy against other solid tumors [171–173]. In addition, surufatinib, which targets CSF-1R, has shown encouraging antitumor activity in the treatment of well-differentiated neuroendocrine tumors. Currently, the phase II trial of surufatinib has been completed, and other two phase III trials are ongoing [174]. The trial of ARRY-382 (CSF-1R inhibitor) combined with pembrolizumab (PD-1 monoclonal antibody) for solid tumors has not demonstrated positive efficacy [175]. In addition, other targets that inhibit recruitment CCL2 and CCL5 have good tolerance but poor anti-tumor activity in clinical trials [121–123, 176].

**Table 3:** Clinical trials of TAMs targeted therapies.

**Targets**	**Drugs**	**Phase**	**Tumor**	**Status**	**Time**	**NCT**
CSF-1	PD-0360324	Ib/II	Advanced Cancer	Active	2015.11.9-present	NCT02554812
CSF-1R	JNJ-40346527	I	Relapsed or Refractory Hodgkin Lymphoma	Completed	2012.7.17-2013.8.13	NCT01572519
		I	Prostate Adenocarcinoma	Active	2017.6.7-present	NCT03177460
		II	Acute Myeloid Leukemia	Terminated	2018.10.5-2020.9.28	NCT03557970
	TPX-0022	I/II	Solid Tumor	Recruiting	2019.9.21-present	NCT03993873
	Cabiralizumab	I	Advanced Solid Tumor	Completed	2015.9.8-2019.11.18	NCT02526017
		II	Advanced Pancreatic Cancer	Active	2017.12.19-present	NCT03336216
		I	Advanced Malignancies	Completed	2017.5.25-2019.10.23	NCT03158272
		I	Cancer	Active	2018.3.15-present	NCT03431948
		I	Advanced Cancer	Completed	2018.5.7-2021.3.31	NCT03335540
		I	Advanced Melanoma and non-small Cell Lung Cancer, renal Cell Carcinoma	Active	2018.6.9-present	NCT03502330
		II	Pancreatic Cancer	Terminated	2018.7.31-2020.6.15	NCT03599362
		II	Pancreatic Cancer	Suspended	2019.10.31-present	NCT03697564
		II	Peripheral T Cell Lymphoma	Active	2019.4.25-present	NCT03927105
		II	Hepatocellular Carcinoma	Active	2019.9.12-present	NCT04050462
		I/II	Triple Negative Breast Cancer	Recruiting	2020.11.19-present	NCT04331067
		II	Head and Neck Squamous Cell Carcinoma	Recruiting	2021.4.24-present	NCT04848116
	IMC-CS4	I	Advanced Solid Tumor	Completed	2011.6.1-2018.5.31	NCT01346358
		I	Pancreatic Cancer	Active	2018.9.27-present	NCT03153410
	SNDX-6352	I	Solid Tumor	Completed	2017.9.1-2020.11.20	NCT03238027
		II	Unresectable Intrahepatic Cholangiocarcinoma	Active	2021.8.24-present	NCT04301778
	BLZ945	I/II	Advanced Solid Tumor	Terminated	2016.10.21-2022.12.1	NCT02829723
	DCC-3014	I/II	Advanced Malignant Neoplasm Pigmented Villonodular Synovitis Giant Cell Tumor of Tendon Sheath	Recruiting	2017.2.16-present	NCT03069469
		III	Tenosynovial Giant Cell Tumor	Recruiting	2021.10.14-present	NCT05059262
	ARRY-382	Ib/II	Solid Tumor	Completed	2016.9-2019.10	NCT02880371
	PLX73086	I	Solid Tumor	Terminated	2016.2.1-2018.1	NCT02673736
	Emactuzumab	Ib	Combine with Atezolizumab in Advanced Solid Tumor	Completed	2015.1.19-2020.8.21	NCT02323191
		Ib	Combine with Selicrelumab in Solid Tumor	Completed	2016.5.9-2018.4.6	NCT02760797
		I	Combine with Paclitaxel in Solid Tumor	Completed	2011.12.20-2018.2.7	NCT01494688
		III	Tenosynovial Giant Cell Tumor	Active	2022.7.18-present	NCT05417789
	PLX3397	III	Tenosynovial Giant Cell Tumor	Completed	2015.5-2016.9	NCT02371369
		II	Recurrent Glioblastoma	Completed	2011.12.3-2013.11.5	NCT01349036
		I/II	Tenosynovial Giant Cell Tumor	Completed	2012.9-2014.4	NCT01004861
		I	Combine with Sirolimus in Unresectable Sarcoma and Malignant Peripheral Nerve Sheath Tumor	Completed	2015.4-2018.9	NCT02584647
		I	Combine with MEK162 in Gastrointestinal Stromal Tumor	Completed	2017.4.15-2021.4.28	NCT03158103
CCR2	MLN1202	II	Metastatic Cancer	Completed	2010.3.1-2012.12	NCT01015560
		I	Melanoma	Terminated	2016.6.22-2018.5.11	NCT02723006
	PF-04136309	II	Metastatic Pancreatic Ductal Adenocarcinoma	Terminated	2016.5.4-2017.9.15	NCT02732938
		I	Pancreatic Neoplasms	Completed	2012.4-2013.10	NCT01413022
	CCX872-B	I	Pancreas Cancer	Completed	2015.2.1-2020.5.6	NCT02345408
CCL2	Carlumab	Ib	Solid Tumor	Completed	2010.5-2010.11	NCT01204996
		II	Metastatic Castration-Resistant Prostate Cancer	Completed	2009.9-2011.11	NCT00992186
CCL5	Maraviroc	I	Colorectal cancer	Completed	2018.5-2018.11	NCT03274804
CXCR4	AMD3100	I	Pediatric Acute Myeloblastic Leukemia and Lymphoblastic Leukemia	Terminated	2012.6-2014.4	NCT01655875
		I/II	Acute Myeloid Leukemia	Completed	2007.7.1-2010.6	NCT00512252
PI3Kγ	BYL719	III	Advanced HER2+ Breast Cancer	Active	2020.7.16-present	NCT04208178
		I	Ovarian Cancer, Breast Cancer	Completed	2012.9.1-2019.5	NCT01623349
	BKM120	I	Carcinoma, Non-Small-Cell Lung	Completed	2013.4.1-2017.10.17	NCT02128724
		II	High Risk Prostate Cancer	Terminated	2013.4.23-2015.2.5	NCT01695473
		I	Breast Cancer	Completed	2012.10.1-2014.2	NCT01513356
TLR-8	Motolimod	Ib	Combine with Cetuximab in Head and Neck Squamous Cell Carcinoma	Completed	2014.10.28-2016.8.11	NCT02124850
TLR-7/8	R848	I	Melanoma (Skin)	Completed	2006.4.1-2011.10	NCT00470379
		I/II	Nodular Basal Cell Carcinoma	Terminated	2013.2.1-2013.8	NCT01808950
CD40	ABBV-428	I	Solid Tumor	Completed	2016.11-2018.6	NCT02955251
	Selicrelumab	I	Resectable Pancreatic Cancer	Completed	2015.10-2018.11	NCT02588443
	Sotigalimab	II	Combine with Nivolumab in Metastatic Pancreatic Cancer	Completed	2018.8-2019.6	NCT03214250
	APX005M	I	Combine with Cabiralizumab with or without Nivolumab in Melanoma, Kidney Cancer, or Non–Small Cell Lung Cancer	Completed	2018.6-2019.4	NCT03502330
	ADC-1013	I/II	Metastatic Pancreatic Ductal Adenocarcinoma	Recruiting	2021.9.17-present	NCT04888312
		I	Solid Tumor	Completed	2015.4.1-2017.3.8	NCT02379741
		I	Solid Tumor	Completed	2015.4-2016.12	NCT02379741
	Chi Lob 7/4	I	Non-Hodgkin Lymphoma	Completed	2007.7.1-2014.10	NCT01561911
CD47	Hu5F9-G4	Ib	Relapsed or Refractory non-Hodgkin’s Lymphoma	Completed	2016.10-2017.11	NCT02953509
	CC-90002	I	Relapsed/Refractory Acute Myeloid Leukemia or High-risk Myelodysplastic Syndromes.	Completed	2016.3-2018.7	NCT02641002
SIRP-α	TTI-621	I	Relapsed or Refractory Hematologic Malignancies	Completed	2016.1.31-2022.12.31	NCT02663518
CD47 and SIRP-α	RRx-001	I	Advanced, Malignant, Incurable Solid Tumor	Completed	2011.10-2013.3	NCT01359982
		I/II	Brain Metastases	Completed	2015.2.6-2016.11.28	NCT02215512
		II	Colorectal Cancer	Completed	2014.5-2018.4.13	NCT02096354

Besides inhibiting the recruitment of TAMs, regulating the repolarization of M2-type TAMs and targeting CD40 have also been tested in clinical trials [177–179]. Duvelisib, a PI3Kγ inhibitor, has shown promising clinical benefits in phase I trials, and the phase II and III trials are being further studied [180, 181]. Clinical responses have been seen across a range of doses and disease subtypes: iNHL overall response rate 58%; relapsed/refractory CLL 56%; peripheral TCL 50%; and cutaneous TCL 32%. In addition, as a dual inhibitor of SIRP-α and CD47, RRx-001 has shown excellent efficacy in the studies of solid tumors such as brain metastases, small cell carcinoma, and colorectal cancer. At present, some phase II trials of RRx-001 have been completed with promising efficacy [182–184]. Hu5F9-G4, TTI-621, and other drugs targeting CD47 have also shown good clinical activity in phase I trials [185–187]. In terms of safety, most of drugs have shown tolerable adverse side effects, with only fatigue, pruritus, headache, nausea, vomiting, edema, and other common adverse reactions reported. In some drug trials targeting CSF-1R, patients showed elevated liver enzymes, which may be related to the destruction of Kupffer cells in the liver [167, 173, 174]. Meanwhile, some clinical trials of hematological tumors and highly malignant endocrine tumors have shown changes in the blood system such as neutropenia and lymphopenia [168, 174, 180]. In addition, the results of phase III clinical trials of PLX3397 have shown obvious hepatotoxicity, which may be related to the expression of CSF-1R in Kupffer cells in the liver [167].

Clinical trials targeting TAMs with positive clinical efficacy have not been observed, and we summarize the reasons as follows. Firstly, the main purpose of phase I trials is to test the safety of drugs, so the groups are often divided by dose escalation, which leads to a small number of cases in the high-dose group to achieve therapeutic effect. Therefore, researchers suggest to increase the dosage of the drugs if patients in clinical trials can tolerate the side effects [173, 175, 188]. Secondly, some patients stopped the trial because of obvious adverse reactions in phase I trials, leading to only a small sample size and no meaningful efficacy evaluation [189]. Therefore, it may be necessary to further increase the sample size or seek means to reduce drug toxicity. Thirdly, some drugs are off-target, so they cannot effectively target TAMs [121–123, 172, 176]. The off-target effect also has an impact on normal immune response, such as infections, autoimmunity, and cancer. Some studies have analyzed that the drug concentration in peripheral blood may be low due to the M2-type TAMs in the skin [172]. Thus, it is necessary to further analyze its pharmacokinetics and pharmacodynamics in order to improve drug development.

## Perspectives

Currently used tumor drug therapy has great limitations, and it is difficult to overcome resistance in long-term application. A large number of clinical studies emphasize the crucial role of the TME and TAMs in immune response, leading to poor clinical response and drug resistance. Preclinical studies have demonstrated that conventional drug therapy reprograms macrophages, improves immunosuppressive effects, and promotes tumor proliferation and metastasis [69, 76, 80]. TAM reprogramming after drug treatment may occur through several mechanisms, including increased TAM recruitment, TAM polarization to the M2-like phenotype, the promotion of pro-angiogenic TAM subsets, and the regulation of TAM proangiogenic activities. In various treatments (chemotherapy, immunotherapy, and targeted therapy), the mechanisms of reprogramed TAMs mediating drug resistance are different. Through extensive research on the interaction between the TME and TAMs, TAM reprogramming, and the mechanism of resistance mediated by TAMs, researchers have developed drugs targeting TAMs and applied them in combination with conventional drug therapy. Reducing or depleting TAMs, repolarizing TAMs M1-like types, enhancing the immune function of T cells, inhibiting the “don’t eat me” signal, and combining with emerging nanomaterials technology, drug resistance was significantly overcome, and the efficacy of traditional drug treatment was improved. Phase I clinical trials have been completed for many drugs, and most of them have good safety without obvious side effects. Although some drugs failed to advance to phase II trials due to poor clinical efficacy and the off-target effect, the reasons for their poor efficacy were analyzed and summarized, and researchers improved some drugs via a combination with biological materials to enhance the drugs. Therefore, the synergy of multiple therapeutic strategies centered on TAMs may further promote the development of cancer therapy, have great development potential and exploration value, and merit further research.

A large number of TAM targeted therapy clinical trials are ongoing, so there are still many challenges that need to be faced. Whether it is necessary to check the number of macrophages in the tumor microenvironment before TAM targeted therapy, and how to select suitable patients are the priorities of treatment. There are multiple mechanisms of targeting TAM therapy, thus the individualized treatment is also worth taking into consideration. During the process of treatment, we should pay attention to the detection of relevant immune indicators. We still need to explore the more reasonable plan of TAM targeted therapy and make it better for clinical translation.

## References

[1] Chen H, Luan X, Paholak HJ, Burnett JP, Stevers NO, Sansanaphongpricha K, He M, Chang AE, Li Q, Sun D. Depleting tumor-associated Tregs via nanoparticle-mediated hyperthermia to enhance anti-CTLA-4 immunotherapy. Nanomedicine (Lond). 2020; 15:77–92. 10.2217/nnm-2019-0190 31868112 PMC7132783

[2] Perdiguero EG, Geissmann F. The development and maintenance of resident macrophages. Nat Immunol. 2016; 17:2–8. 10.1038/ni.3341 26681456 PMC4950995

[3] Ruffell B, Coussens LM. Macrophages and therapeutic resistance in cancer. Cancer Cell. 2015; 27:462–72. 10.1016/j.ccell.2015.02.015 25858805 PMC4400235

[4] Boutilier AJ, Elsawa SF. Macrophage Polarization States in the Tumor Microenvironment. Int J Mol Sci. 2021; 22:6995. 10.3390/ijms22136995 34209703 PMC8268869

[5] Pathria P, Louis TL, Varner JA. Targeting Tumor-Associated Macrophages in Cancer. Trends Immunol. 2019; 40:310–27. 10.1016/j.it.2019.02.003 30890304

[6] Cotechini T, Atallah A, Grossman A. Tissue-Resident and Recruited Macrophages in Primary Tumor and Metastatic Microenvironments: Potential Targets in Cancer Therapy. Cells. 2021; 10:960. 10.3390/cells10040960 33924237 PMC8074766

[7] Balkwill FR, Capasso M, Hagemann T. The tumor microenvironment at a glance. J Cell Sci. 2012; 125:5591–6. 10.1242/jcs.116392 23420197

[8] Suarez-Lopez L, Sriram G, Kong YW, Morandell S, Merrick KA, Hernandez Y, Haigis KM, Yaffe MB. MK2 contributes to tumor progression by promoting M2 macrophage polarization and tumor angiogenesis. Proc Natl Acad Sci U S A. 2018; 115:E4236–44. 10.1073/pnas.1722020115 29666270 PMC5939099

[9] Kuziel G, Thompson V, D'Amato JV, Arendt LM. Stromal CCL2 Signaling Promotes Mammary Tumor Fibrosis through Recruitment of Myeloid-Lineage Cells. Cancers (Basel). 2020; 12:2083. 10.3390/cancers12082083 32731354 PMC7465971

[10] Loyher PL, Hamon P, Laviron M, Meghraoui-Kheddar A, Goncalves E, Deng Z, Torstensson S, Bercovici N, Baudesson de Chanville C, Combadière B, Geissmann F, Savina A, Combadière C, Boissonnas A. Macrophages of distinct origins contribute to tumor development in the lung. J Exp Med. 2018; 215:2536–53. 10.1084/jem.20180534 30201786 PMC6170177

[11] Wyckoff J, Wang W, Lin EY, Wang Y, Pixley F, Stanley ER, Graf T, Pollard JW, Segall J, Condeelis J. A paracrine loop between tumor cells and macrophages is required for tumor cell migration in mammary tumors. Cancer Res. 2004; 64:7022–9. 10.1158/0008-5472.CAN-04-1449 15466195

[12] Wyckoff JB, Wang Y, Lin EY, Li JF, Goswami S, Stanley ER, Segall JE, Pollard JW, Condeelis J. Direct visualization of macrophage-assisted tumor cell intravasation in mammary tumors. Cancer Res. 2007; 67:2649–56. 10.1158/0008-5472.CAN-06-1823 17363585

[13] Bronte V, Brandau S, Chen SH, Colombo MP, Frey AB, Greten TF, Mandruzzato S, Murray PJ, Ochoa A, Ostrand-Rosenberg S, Rodriguez PC, Sica A, Umansky V, et al. Recommendations for myeloid-derived suppressor cell nomenclature and characterization standards. Nat Commun. 2016; 7:12150. 10.1038/ncomms12150 27381735 PMC4935811

[14] Lewis CE, Harney AS, Pollard JW. The Multifaceted Role of Perivascular Macrophages in Tumors. Cancer Cell. 2016; 30:18–25. 10.1016/j.ccell.2016.05.017 27411586 PMC5024543

[15] Harney AS, Arwert EN, Entenberg D, Wang Y, Guo P, Qian BZ, Oktay MH, Pollard JW, Jones JG, Condeelis JS. Real-Time Imaging Reveals Local, Transient Vascular Permeability, and Tumor Cell Intravasation Stimulated by TIE2hi Macrophage-Derived VEGFA. Cancer Discov. 2015; 5:932–43. 10.1158/2159-8290.CD-15-0012 26269515 PMC4560669

[16] Korbecki J, Kojder K, Kapczuk P, Kupnicka P, Gawrońska-Szklarz B, Gutowska I, Chlubek D, Baranowska-Bosiacka I. The Effect of Hypoxia on the Expression of CXC Chemokines and CXC Chemokine Receptors-A Review of Literature. Int J Mol Sci. 2021; 22:843. 10.3390/ijms22020843 33467722 PMC7830156

[17] Cárdenas-Navia LI, Mace D, Richardson RA, Wilson DF, Shan S, Dewhirst MW. The pervasive presence of fluctuating oxygenation in tumors. Cancer Res. 2008; 68:5812–9. 10.1158/0008-5472.CAN-07-6387 18632635

[18] Victor N, Ivy A, Jiang BH, Agani FH. Involvement of HIF-1 in invasion of Mum2B uveal melanoma cells. Clin Exp Metastasis. 2006; 23:87–96. 10.1007/s10585-006-9024-z 16826425

[19] Konishi H, Shirabe K, Nakagawara H, Harimoto N, Yamashita Y, Ikegami T, Yoshizumi T, Soejima Y, Oda Y, Maehara Y. Suppression of silent information regulator 1 activity in noncancerous tissues of hepatocellular carcinoma: Possible association with non-B non-C hepatitis pathogenesis. Cancer Sci. 2015; 106:542–9. 10.1111/cas.12653 25736100 PMC4452154

[20] Ye LY, Chen W, Bai XL, Xu XY, Zhang Q, Xia XF, Sun X, Li GG, Hu QD, Fu QH, Liang TB. Hypoxia-Induced Epithelial-to-Mesenchymal Transition in Hepatocellular Carcinoma Induces an Immunosuppressive Tumor Microenvironment to Promote Metastasis. Cancer Res. 2016; 76:818–30. 10.1158/0008-5472.CAN-15-0977 26837767

[21] Fang HY, Hughes R, Murdoch C, Coffelt SB, Biswas SK, Harris AL, Johnson RS, Imityaz HZ, Simon MC, Fredlund E, Greten FR, Rius J, Lewis CE. Hypoxia-inducible factors 1 and 2 are important transcriptional effectors in primary macrophages experiencing hypoxia. Blood. 2009; 114:844–59. 10.1182/blood-2008-12-195941 19454749 PMC2882173

[22] Korbecki J, Simińska D, Gąssowska-Dobrowolska M, Listos J, Gutowska I, Chlubek D, Baranowska-Bosiacka I. Chronic and Cycling Hypoxia: Drivers of Cancer Chronic Inflammation through HIF-1 and NF-κB Activation: A Review of the Molecular Mechanisms. Int J Mol Sci. 2021; 22:10701. 10.3390/ijms221910701 34639040 PMC8509318

[23] Ke X, Chen C, Song Y, Cai Q, Li J, Tang Y, Han X, Qu W, Chen A, Wang H, Xu G, Liu D. Hypoxia modifies the polarization of macrophages and their inflammatory microenvironment, and inhibits malignant behavior in cancer cells. Oncol Lett. 2019; 18:5871–8. 10.3892/ol.2019.10956 31788060 PMC6865149

[24] Colegio OR, Chu NQ, Szabo AL, Chu T, Rhebergen AM, Jairam V, Cyrus N, Brokowski CE, Eisenbarth SC, Phillips GM, Cline GW, Phillips AJ, Medzhitov R. Functional polarization of tumour-associated macrophages by tumour-derived lactic acid. Nature. 2014; 513:559–63. 10.1038/nature13490 25043024 PMC4301845

[25] Scott DW, Chan FC, Hong F, Rogic S, Tan KL, Meissner B, Ben-Neriah S, Boyle M, Kridel R, Telenius A, Woolcock BW, Farinha P, Fisher RI, et al. Gene expression-based model using formalin-fixed paraffin-embedded biopsies predicts overall survival in advanced-stage classical Hodgkin lymphoma. J Clin Oncol. 2013; 31:692–700. 10.1200/JCO.2012.43.4589 23182984 PMC3574267

[26] Blenman KRM, Marczyk M, Karn T, Qing T, Li X, Gunasekharan V, Yaghoobi V, Bai Y, Ibrahim EY, Park T, Silber A, Wolf DM, Reisenbichler E, et al. Predictive Markers of Response to Neoadjuvant Durvalumab with Nab-Paclitaxel and Dose-Dense Doxorubicin/Cyclophosphamide in Basal-Like Triple-Negative Breast Cancer. Clin Cancer Res. 2022; 28:2587–97. 10.1158/1078-0432.CCR-21-3215 35377948 PMC9464605

[27] Shah MA, Enzinger P, Ko AH, Ocean AJ, Philip PA, Thakkar PV, Cleveland K, Lu Y, Kortmansky J, Christos PJ, Zhang C, Kaur N, Elmonshed D, et al. Multicenter Phase II Study of Cabazitaxel in Advanced Gastroesophageal Cancer: Association of HER2 Expression and M2-Like Tumor-Associated Macrophages with Patient Outcome. Clin Cancer Res. 2020; 26:4756–66. 10.1158/1078-0432.CCR-19-3920 32641434 PMC8209413

[28] McLemore LE, Janakiram M, Albanese J, Shapiro N, Lo Y, Zang X, Fineberg S. An Immunoscore Using PD-L1, CD68, and Tumor-infiltrating Lymphocytes (TILs) to Predict Response to Neoadjuvant Chemotherapy in Invasive Breast Cancer. Appl Immunohistochem Mol Morphol. 2018; 26:611–9. 10.1097/PAI.0000000000000485 28422766

[29] Riihijärvi S, Fiskvik I, Taskinen M, Vajavaara H, Tikkala M, Yri O, Karjalainen-Lindsberg ML, Delabie J, Smeland E, Holte H, Leppä S. Prognostic influence of macrophages in patients with diffuse large B-cell lymphoma: a correlative study from a Nordic phase II trial. Haematologica. 2015; 100:238–45. 10.3324/haematol.2014.113472 25381134 PMC4803141

[30] Sun BY, Zhou C, Guan RY, Liu G, Yang ZF, Wang ZT, Gan W, Zhou J, Fan J, Yi Y, Qiu SJ. Dissecting Intra-Tumoral Changes Following Immune Checkpoint Blockades in Intrahepatic Cholangiocarcinoma via Single-Cell Analysis. Front Immunol. 2022; 13:871769. 10.3389/fimmu.2022.871769 35558087 PMC9088915

[31] Toulmonde M, Penel N, Adam J, Chevreau C, Blay JY, Le Cesne A, Bompas E, Piperno-Neumann S, Cousin S, Grellety T, Ryckewaert T, Bessede A, Ghiringhelli F, et al. Use of PD-1 Targeting, Macrophage Infiltration, and IDO Pathway Activation in Sarcomas: A Phase 2 Clinical Trial. JAMA Oncol. 2018; 4:93–7. 10.1001/jamaoncol.2017.1617 28662235 PMC5833654

[32] Augustyn A, Adams DL, He J, Qiao Y, Verma V, Liao Z, Tang CM, Heymach JV, Tsao AS, Lin SH. Giant Circulating Cancer-Associated Macrophage-Like Cells Are Associated With Disease Recurrence and Survival in Non-Small-Cell Lung Cancer Treated With Chemoradiation and Atezolizumab. Clin Lung Cancer. 2021; 22:e451–65. 10.1016/j.cllc.2020.06.016 32798130

[33] Jiang T, Wang P, Zhang J, Zhao Y, Zhou J, Fan Y, Shu Y, Liu X, Zhang H, He J, Gao G, Mu X, Bao Z, et al. Toripalimab plus chemotherapy as second-line treatment in previously EGFR-TKI treated patients with EGFR-mutant-advanced NSCLC: a multicenter phase-II trial. Signal Transduct Target Ther. 2021; 6:355. 10.1038/s41392-021-00751-9 34650034 PMC8517012

[34] Ma X, Guo Z, Wei X, Zhao G, Han D, Zhang T, Chen X, Cao F, Dong J, Zhao L, Yuan Z, Wang P, Pang Q, et al. Spatial Distribution and Predictive Significance of Dendritic Cells and Macrophages in Esophageal Cancer Treated With Combined Chemoradiotherapy and PD-1 Blockade. Front Immunol. 2021; 12:786429. 10.3389/fimmu.2021.786429 35046943 PMC8761740

[35] Hodi FS, Lawrence D, Lezcano C, Wu X, Zhou J, Sasada T, Zeng W, Giobbie-Hurder A, Atkins MB, Ibrahim N, Friedlander P, Flaherty KT, Murphy GF, et al. Bevacizumab plus ipilimumab in patients with metastatic melanoma. Cancer Immunol Res. 2014; 2:632–42. 10.1158/2326-6066.CIR-14-0053 24838938 PMC4306338

[36] Yan ZX, Li L, Wang W, OuYang BS, Cheng S, Wang L, Wu W, Xu PP, Muftuoglu M, Hao M, Yang S, Zhang MC, Zheng Z, et al. Clinical Efficacy and Tumor Microenvironment Influence in a Dose-Escalation Study of Anti-CD19 Chimeric Antigen Receptor T Cells in Refractory B-Cell Non-Hodgkin's Lymphoma. Clin Cancer Res. 2019; 25:6995–7003. 10.1158/1078-0432.CCR-19-0101 31444250

[37] Jayson GC, Kerbel R, Ellis LM, Harris AL. Antiangiogenic therapy in oncology: current status and future directions. Lancet. 2016; 388:518–29. 10.1016/S0140-6736(15)01088-0 26853587

[38] Castro BA, Flanigan P, Jahangiri A, Hoffman D, Chen W, Kuang R, De Lay M, Yagnik G, Wagner JR, Mascharak S, Sidorov M, Shrivastav S, Kohanbash G, et al. Macrophage migration inhibitory factor downregulation: a novel mechanism of resistance to anti-angiogenic therapy. Oncogene. 2017; 36:3749–59. 10.1038/onc.2017.1 28218903 PMC5491354

[39] Gabrusiewicz K, Liu D, Cortes-Santiago N, Hossain MB, Conrad CA, Aldape KD, Fuller GN, Marini FC, Alonso MM, Idoate MA, Gilbert MR, Fueyo J, Gomez-Manzano C. Anti-vascular endothelial growth factor therapy-induced glioma invasion is associated with accumulation of Tie2-expressing monocytes. Oncotarget. 2014; 5:2208–20. 10.18632/oncotarget.1893 24809734 PMC4039157

[40] Lu-Emerson C, Snuderl M, Kirkpatrick ND, Goveia J, Davidson C, Huang Y, Riedemann L, Taylor J, Ivy P, Duda DG, Ancukiewicz M, Plotkin SR, Chi AS, et al. Increase in tumor-associated macrophages after antiangiogenic therapy is associated with poor survival among patients with recurrent glioblastoma. Neuro Oncol. 2013; 15:1079–87. 10.1093/neuonc/not082 23828240 PMC3714160

[41] Zhu C, Kros JM, Cheng C, Mustafa D. The contribution of tumor-associated macrophages in glioma neo-angiogenesis and implications for anti-angiogenic strategies. Neuro Oncol. 2017; 19:1435–46. 10.1093/neuonc/nox081 28575312 PMC5737221

[42] Xu L, Duda DG, di Tomaso E, Ancukiewicz M, Chung DC, Lauwers GY, Samuel R, Shellito P, Czito BG, Lin PC, Poleski M, Bentley R, Clark JW, et al. Direct evidence that bevacizumab, an anti-VEGF antibody, up-regulates SDF1alpha, CXCR4, CXCL6, and neuropilin 1 in tumors from patients with rectal cancer. Cancer Res. 2009; 69:7905–10. 10.1158/0008-5472.CAN-09-2099 19826039 PMC2859041

[43] Ebos JM, Kerbel RS. Antiangiogenic therapy: impact on invasion, disease progression, and metastasis. Nat Rev Clin Oncol. 2011; 8:210–21. 10.1038/nrclinonc.2011.21 21364524 PMC4540336

[44] Feng PH, Yu CT, Chen KY, Luo CS, Wu SM, Liu CY, Kuo LW, Chan YF, Chen TT, Chang CC, Lee CN, Chuang HC, Lin CF, et al. S100A9^+^ MDSC and TAM-mediated EGFR-TKI resistance in lung adenocarcinoma: the role of *RELB*. Oncotarget. 2018; 9:7631–43. 10.18632/oncotarget.24146 29484139 PMC5800931

[45] Noy R, Pollard JW. Tumor-associated macrophages: from mechanisms to therapy. Immunity. 2014; 41:49–61. 10.1016/j.immuni.2014.06.010 25035953 PMC4137410

[46] Ostuni R, Kratochvill F, Murray PJ, Natoli G. Macrophages and cancer: from mechanisms to therapeutic implications. Trends Immunol. 2015; 36:229–39. 10.1016/j.it.2015.02.004 25770924

[47] Chung AS, Wu X, Zhuang G, Ngu H, Kasman I, Zhang J, Vernes JM, Jiang Z, Meng YG, Peale FV, Ouyang W, Ferrara N. An interleukin-17-mediated paracrine network promotes tumor resistance to anti-angiogenic therapy. Nat Med. 2013; 19:1114–23. 10.1038/nm.3291 23913124

[48] Shojaei F, Wu X, Malik AK, Zhong C, Baldwin ME, Schanz S, Fuh G, Gerber HP, Ferrara N. Tumor refractoriness to anti-VEGF treatment is mediated by CD11b+Gr1+ myeloid cells. Nat Biotechnol. 2007; 25:911–20. 10.1038/nbt1323 17664940

[49] Shojaei F, Wu X, Zhong C, Yu L, Liang XH, Yao J, Blanchard D, Bais C, Peale FV, van Bruggen N, Ho C, Ross J, Tan M, et al. Bv8 regulates myeloid-cell-dependent tumour angiogenesis. Nature. 2007; 450:825–31. 10.1038/nature06348 18064003

[50] Kadioglu E, De Palma M. Cancer Metastasis: Perivascular Macrophages Under Watch. Cancer Discov. 2015; 5:906–8. 10.1158/2159-8290.CD-15-0819 26334045

[51] Cho HR, Kumari N, Thi Vu H, Kim H, Park CK, Choi SH. Increased Antiangiogenic Effect by Blocking CCL2-dependent Macrophages in a Rodent Glioblastoma Model: Correlation Study with Dynamic Susceptibility Contrast Perfusion MRI. Sci Rep. 2019; 9:11085. 10.1038/s41598-019-47438-4 31366997 PMC6668454

[52] Qian BZ, Li J, Zhang H, Kitamura T, Zhang J, Campion LR, Kaiser EA, Snyder LA, Pollard JW. CCL2 recruits inflammatory monocytes to facilitate breast-tumour metastasis. Nature. 2011; 475:222–5. 10.1038/nature10138 21654748 PMC3208506

[53] Zeisberger SM, Odermatt B, Marty C, Zehnder-Fjällman AH, Ballmer-Hofer K, Schwendener RA. Clodronate-liposome-mediated depletion of tumour-associated macrophages: a new and highly effective antiangiogenic therapy approach. Br J Cancer. 2006; 95:272–81. 10.1038/sj.bjc.6603240 16832418 PMC2360657

[54] Zhang W, Zhu XD, Sun HC, Xiong YQ, Zhuang PY, Xu HX, Kong LQ, Wang L, Wu WZ, Tang ZY. Depletion of tumor-associated macrophages enhances the effect of sorafenib in metastatic liver cancer models by antimetastatic and antiangiogenic effects. Clin Cancer Res. 2010; 16:3420–30. 10.1158/1078-0432.CCR-09-2904 20570927

[55] Chung FT, Lee KY, Wang CW, Heh CC, Chan YF, Chen HW, Kuo CH, Feng PH, Lin TY, Wang CH, Chou CL, Chen HC, Lin SM, Kuo HP. Tumor-associated macrophages correlate with response to epidermal growth factor receptor-tyrosine kinase inhibitors in advanced non-small cell lung cancer. Int J Cancer. 2012; 131:E227–35. 10.1002/ijc.27403 22174092

[56] Sunakawa Y, Stintzing S, Cao S, Heinemann V, Cremolini C, Falcone A, Yang D, Zhang W, Ning Y, Stremitzer S, Matsusaka S, Yamauchi S, Parekh A, et al. Variations in genes regulating tumor-associated macrophages (TAMs) to predict outcomes of bevacizumab-based treatment in patients with metastatic colorectal cancer: results from TRIBE and FIRE3 trials. Ann Oncol. 2015; 26:2450–6. 10.1093/annonc/mdv474 26416897 PMC4658546

[57] Zhang B, Zhang Y, Zhao J, Wang Z, Wu T, Ou W, Wang J, Yang B, Zhao Y, Rao Z, Gao J. M2-polarized macrophages contribute to the decreased sensitivity of EGFR-TKIs treatment in patients with advanced lung adenocarcinoma. Med Oncol. 2014; 31:127. 10.1007/s12032-014-0127-0 25034365

[58] Innocenti F, Yazdani A, Rashid N, Qu X, Ou FS, Van Buren S, Bertagnolli MM, Kabbarah O, Blanke CD, Venook AP, Lenz HJ, Vincent BG. Tumor Immunogenomic Features Determine Outcomes in Patients with Metastatic Colorectal Cancer Treated with Standard-of-Care Combinations of Bevacizumab and Cetuximab. Clin Cancer Res. 2022; 28:1690–700. 10.1158/1078-0432.CCR-21-3202 35176136 PMC9093780

[59] Pander J, Heusinkveld M, van der Straaten T, Jordanova ES, Baak-Pablo R, Gelderblom H, Morreau H, van der Burg SH, Guchelaar HJ, van Hall T. Activation of tumor-promoting type 2 macrophages by EGFR-targeting antibody cetuximab. Clin Cancer Res. 2011; 17:5668–73. 10.1158/1078-0432.CCR-11-0239 21788356

[60] Choueiri TK, Figueroa DJ, Fay AP, Signoretti S, Liu Y, Gagnon R, Deen K, Carpenter C, Benson P, Ho TH, Pandite L, de Souza P, Powles T, Motzer RJ. Correlation of PD-L1 tumor expression and treatment outcomes in patients with renal cell carcinoma receiving sunitinib or pazopanib: results from COMPARZ, a randomized controlled trial. Clin Cancer Res. 2015; 21:1071–7. 10.1158/1078-0432.CCR-14-1993 25538263

[61] Hakimi AA, Voss MH, Kuo F, Sanchez A, Liu M, Nixon BG, Vuong L, Ostrovnaya I, Chen YB, Reuter V, Riaz N, Cheng Y, Patel P, et al. Transcriptomic Profiling of the Tumor Microenvironment Reveals Distinct Subgroups of Clear Cell Renal Cell Cancer: Data from a Randomized Phase III Trial. Cancer Discov. 2019; 9:510–25. 10.1158/2159-8290.CD-18-0957 30622105 PMC6697163

[62] Conley SJ, Gheordunescu E, Kakarala P, Newman B, Korkaya H, Heath AN, Clouthier SG, Wicha MS. Antiangiogenic agents increase breast cancer stem cells via the generation of tumor hypoxia. Proc Natl Acad Sci U S A. 2012; 109:2784–9. 10.1073/pnas.1018866109 22308314 PMC3286974

[63] De Bock K, Mazzone M, Carmeliet P. Antiangiogenic therapy, hypoxia, and metastasis: risky liaisons, or not? Nat Rev Clin Oncol. 2011; 8:393–404. 10.1038/nrclinonc.2011.83 21629216

[64] Jain RK. Antiangiogenesis strategies revisited: from starving tumors to alleviating hypoxia. Cancer Cell. 2014; 26:605–22. 10.1016/j.ccell.2014.10.006 25517747 PMC4269830

[65] Miyazaki S, Kikuchi H, Iino I, Uehara T, Setoguchi T, Fujita T, Hiramatsu Y, Ohta M, Kamiya K, Kitagawa K, Kitagawa M, Baba S, Konno H. Anti-VEGF antibody therapy induces tumor hypoxia and stanniocalcin 2 expression and potentiates growth of human colon cancer xenografts. Int J Cancer. 2014; 135:295–307. 10.1002/ijc.28686 24375080

[66] Henze AT, Mazzone M. The impact of hypoxia on tumor-associated macrophages. J Clin Invest. 2016; 126:3672–9. 10.1172/JCI84427 27482883 PMC5096805

[67] Keith B, Johnson RS, Simon MC. HIF1α and HIF2α: sibling rivalry in hypoxic tumour growth and progression. Nat Rev Cancer. 2011; 12:9–22. 10.1038/nrc3183 22169972 PMC3401912

[68] LaGory EL, Giaccia AJ. The ever-expanding role of HIF in tumour and stromal biology. Nat Cell Biol. 2016; 18:356–65. 10.1038/ncb3330 27027486 PMC4898054

[69] Murdoch C, Giannoudis A, Lewis CE. Mechanisms regulating the recruitment of macrophages into hypoxic areas of tumors and other ischemic tissues. Blood. 2004; 104:2224–34. 10.1182/blood-2004-03-1109 15231578

[70] Deshmukh SK, Tyagi N, Khan MA, Srivastava SK, Al-Ghadhban A, Dugger K, Carter JE, Singh S, Singh AP. Gemcitabine treatment promotes immunosuppressive microenvironment in pancreatic tumors by supporting the infiltration, growth, and polarization of macrophages. Sci Rep. 2018; 8:12000. 10.1038/s41598-018-30437-2 30097594 PMC6086900

[71] Liu T, Larionova I, Litviakov N, Riabov V, Zavyalova M, Tsyganov M, Buldakov M, Song B, Moganti K, Kazantseva P, Slonimskaya E, Kremmer E, Flatley A, et al. Tumor-associated macrophages in human breast cancer produce new monocyte attracting and pro-angiogenic factor YKL-39 indicative for increased metastasis after neoadjuvant chemotherapy. Oncoimmunology. 2018; 7:e1436922. 10.1080/2162402X.2018.1436922 29872578 PMC5980380

[72] Wesolowski R, Sharma N, Reebel L, Rodal MB, Peck A, West BL, Marimuthu A, Severson P, Karlin DA, Dowlati A, Le MH, Coussens LM, Rugo HS. Phase Ib study of the combination of pexidartinib (PLX3397), a CSF-1R inhibitor, and paclitaxel in patients with advanced solid tumors. Ther Adv Med Oncol. 2019; 11:1758835919854238. 10.1177/1758835919854238 31258629 PMC6589951

[73] Bhattacharya U, Gutter-Kapon L, Kan T, Boyango I, Barash U, Yang SM, Liu J, Gross-Cohen M, Sanderson RD, Shaked Y, Ilan N, Vlodavsky I. Heparanase and Chemotherapy Synergize to Drive Macrophage Activation and Enhance Tumor Growth. Cancer Res. 2020; 80:57–68. 10.1158/0008-5472.CAN-19-1676 31690669 PMC6942624

[74] Piao Y, Liang J, Holmes L, Zurita AJ, Henry V, Heymach JV, de Groot JF. Glioblastoma resistance to anti-VEGF therapy is associated with myeloid cell infiltration, stem cell accumulation, and a mesenchymal phenotype. Neuro Oncol. 2012; 14:1379–92. 10.1093/neuonc/nos158 22965162 PMC3480262

[75] Tripathi C, Tewari BN, Kanchan RK, Baghel KS, Nautiyal N, Shrivastava R, Kaur H, Bhatt ML, Bhadauria S. Macrophages are recruited to hypoxic tumor areas and acquire a pro-angiogenic M2-polarized phenotype via hypoxic cancer cell derived cytokines Oncostatin M and Eotaxin. Oncotarget. 2014; 5:5350–68. 10.18632/oncotarget.2110 25051364 PMC4170629

[76] Zhou SL, Zhou ZJ, Hu ZQ, Huang XW, Wang Z, Chen EB, Fan J, Cao Y, Dai Z, Zhou J. Tumor-Associated Neutrophils Recruit Macrophages and T-Regulatory Cells to Promote Progression of Hepatocellular Carcinoma and Resistance to Sorafenib. Gastroenterology. 2016; 150:1646–58.e17. 10.1053/j.gastro.2016.02.040 26924089

[77] Wang L, Li YS, Yu LG, Zhang XK, Zhao L, Gong FL, Yang XX, Guo XL. Galectin-3 expression and secretion by tumor-associated macrophages in hypoxia promotes breast cancer progression. Biochem Pharmacol. 2020; 178:114113. 10.1016/j.bcp.2020.114113 32579956

[78] Ngambenjawong C, Gustafson HH, Pun SH. Progress in tumor-associated macrophage (TAM)-targeted therapeutics. Adv Drug Deliv Rev. 2017; 114:206–21. 10.1016/j.addr.2017.04.010 28449873 PMC5581987

[79] de Groot J, Liang J, Kong LY, Wei J, Piao Y, Fuller G, Qiao W, Heimberger AB. Modulating antiangiogenic resistance by inhibiting the signal transducer and activator of transcription 3 pathway in glioblastoma. Oncotarget. 2012; 3:1036–48. 10.18632/oncotarget.663 23013619 PMC3660053

[80] Rivera LB, Meyronet D, Hervieu V, Frederick MJ, Bergsland E, Bergers G. Intratumoral myeloid cells regulate responsiveness and resistance to antiangiogenic therapy. Cell Rep. 2015; 11:577–91. 10.1016/j.celrep.2015.03.055 25892230 PMC4438771

[81] Dijkgraaf EM, Heusinkveld M, Tummers B, Vogelpoel LT, Goedemans R, Jha V, Nortier JW, Welters MJ, Kroep JR, van der Burg SH. Chemotherapy alters monocyte differentiation to favor generation of cancer-supporting M2 macrophages in the tumor microenvironment. Cancer Res. 2013; 73:2480–92. 10.1158/0008-5472.CAN-12-3542 23436796

[82] Roux C, Jafari SM, Shinde R, Duncan G, Cescon DW, Silvester J, Chu MF, Hodgson K, Berger T, Wakeham A, Palomero L, Garcia-Valero M, Pujana MA, et al. Reactive oxygen species modulate macrophage immunosuppressive phenotype through the up-regulation of PD-L1. Proc Natl Acad Sci U S A. 2019; 116:4326–35. 10.1073/pnas.1819473116 30770442 PMC6410837

[83] Chen Z, Huang Y, Hu Z, Zhao M, Bian Y, Chen Z, Zheng Y, Bi G, Pang Y, Zhan C, Lin Z, Guo W, Wang Q, Tan L. Dissecting the single-cell transcriptome network in patients with esophageal squamous cell carcinoma receiving operative paclitaxel plus platinum chemotherapy. Oncogenesis. 2021; 10:71. 10.1038/s41389-021-00359-2 34697289 PMC8546051

[84] Andrade LNS, Otake AH, Cardim SGB, da Silva FI, Ikoma Sakamoto MM, Furuya TK, Uno M, Pasini FS, Chammas R. Extracellular Vesicles Shedding Promotes Melanoma Growth in Response to Chemotherapy. Sci Rep. 2019; 9:14482. 10.1038/s41598-019-50848-z 31597943 PMC6785560

[85] Mimeault M, Batra SK. Hypoxia-inducing factors as master regulators of stemness properties and altered metabolism of cancer- and metastasis-initiating cells. J Cell Mol Med. 2013; 17:30–54. 10.1111/jcmm.12004 23301832 PMC3560853

[86] Dong N, Shi X, Wang S, Gao Y, Kuang Z, Xie Q, Li Y, Deng H, Wu Y, Li M, Li JL. M2 macrophages mediate sorafenib resistance by secreting HGF in a feed-forward manner in hepatocellular carcinoma. Br J Cancer. 2019; 121:22–33. 10.1038/s41416-019-0482-x 31130723 PMC6738111

[87] Cascone T, Heymach JV. Targeting the angiopoietin/Tie2 pathway: cutting tumor vessels with a double-edged sword? J Clin Oncol. 2012; 30:441–4. 10.1200/JCO.2011.38.7621 22184396

[88] Huang H, Bhat A, Woodnutt G, Lappe R. Targeting the ANGPT-TIE2 pathway in malignancy. Nat Rev Cancer. 2010; 10:575–85. 10.1038/nrc2894 20651738

[89] Coffelt SB, Tal AO, Scholz A, De Palma M, Patel S, Urbich C, Biswas SK, Murdoch C, Plate KH, Reiss Y, Lewis CE. Angiopoietin-2 regulates gene expression in TIE2-expressing monocytes and augments their inherent proangiogenic functions. Cancer Res. 2010; 70:5270–80. 10.1158/0008-5472.CAN-10-0012 20530679

[90] Hughes R, Qian BZ, Rowan C, Muthana M, Keklikoglou I, Olson OC, Tazzyman S, Danson S, Addison C, Clemons M, Gonzalez-Angulo AM, Joyce JA, De Palma M, et al. Perivascular M2 Macrophages Stimulate Tumor Relapse after Chemotherapy. Cancer Res. 2015; 75:3479–91. 10.1158/0008-5472.CAN-14-3587 26269531 PMC5024531

[91] Fu XH, Li J, Zou Y, Hong YR, Fu ZX, Huang JJ, Zhang SZ, Zheng S. Endostar enhances the antineoplastic effects of combretastatin A4 phosphate in an osteosarcoma xenograft. Cancer Lett. 2011; 312:109–16. 10.1016/j.canlet.2011.08.008 21893381

[92] Rustin GJ, Galbraith SM, Anderson H, Stratford M, Folkes LK, Sena L, Gumbrell L, Price PM. Phase I clinical trial of weekly combretastatin A4 phosphate: clinical and pharmacokinetic results. J Clin Oncol. 2003; 21:2815–22. 10.1200/JCO.2003.05.185 12807934

[93] Nathan P, Zweifel M, Padhani AR, Koh DM, Ng M, Collins DJ, Harris A, Carden C, Smythe J, Fisher N, Taylor NJ, Stirling JJ, Lu SP, et al. Phase I trial of combretastatin A4 phosphate (CA4P) in combination with bevacizumab in patients with advanced cancer. Clin Cancer Res. 2012; 18:3428–39. 10.1158/1078-0432.CCR-11-3376 22645052

[94] Welford AF, Biziato D, Coffelt SB, Nucera S, Fisher M, Pucci F, Di Serio C, Naldini L, De Palma M, Tozer GM, Lewis CE. TIE2-expressing macrophages limit the therapeutic efficacy of the vascular-disrupting agent combretastatin A4 phosphate in mice. J Clin Invest. 2011; 121:1969–73. 10.1172/JCI44562 21490397 PMC3083764

[95] Steinberger KJ, Forget MA, Bobko AA, Mihalik NE, Gencheva M, Roda JM, Cole SL, Mo X, Hoblitzell EH, Evans R, Gross AC, Moldovan L, Marsh CB, et al. Hypoxia-Inducible Factor α Subunits Regulate Tie2-Expressing Macrophages That Influence Tumor Oxygen and Perfusion in Murine Breast Cancer. J Immunol. 2020; 205:2301–11. 10.4049/jimmunol.2000185 32938724 PMC7596922

[96] Cortes-Santiago N, Hossain MB, Gabrusiewicz K, Fan X, Gumin J, Marini FC, Alonso MM, Lang F, Yung WK, Fueyo J, Gomez-Manzano C. Soluble Tie2 overrides the heightened invasion induced by anti-angiogenesis therapies in gliomas. Oncotarget. 2016; 7:16146–57. 10.18632/oncotarget.7550 26910374 PMC4941303

[97] Coffelt SB, Chen YY, Muthana M, Welford AF, Tal AO, Scholz A, Plate KH, Reiss Y, Murdoch C, De Palma M, Lewis CE. Angiopoietin 2 stimulates TIE2-expressing monocytes to suppress T cell activation and to promote regulatory T cell expansion. J Immunol. 2011; 186:4183–90. 10.4049/jimmunol.1002802 21368233

[98] Mortara L, Benest AV, Bates DO, Noonan DM. Can the co-dependence of the immune system and angiogenesis facilitate pharmacological targeting of tumours? Curr Opin Pharmacol. 2017; 35:66–74. 10.1016/j.coph.2017.05.009 28623714

[99] Ramjiawan RR, Griffioen AW, Duda DG. Anti-angiogenesis for cancer revisited: Is there a role for combinations with immunotherapy? Angiogenesis. 2017; 20:185–204. 10.1007/s10456-017-9552-y 28361267 PMC5439974

[100] Dalton HJ, Pradeep S, McGuire M, Hailemichael Y, Ma S, Lyons Y, Armaiz-Pena GN, Previs RA, Hansen JM, Rupaimoole R, Gonzalez-Villasana V, Cho MS, Wu SY, et al. Macrophages Facilitate Resistance to Anti-VEGF Therapy by Altered VEGFR Expression. Clin Cancer Res. 2017; 23:7034–46. 10.1158/1078-0432.CCR-17-0647 28855350 PMC5690831

[101] Mazzieri R, Pucci F, Moi D, Zonari E, Ranghetti A, Berti A, Politi LS, Gentner B, Brown JL, Naldini L, De Palma M. Targeting the ANG2/TIE2 axis inhibits tumor growth and metastasis by impairing angiogenesis and disabling rebounds of proangiogenic myeloid cells. Cancer Cell. 2011; 19:512–26. 10.1016/j.ccr.2011.02.005 21481792

[102] Meng YM, Liang J, Wu C, Xu J, Zeng DN, Yu XJ, Ning H, Xu L, Zheng L. Monocytes/Macrophages promote vascular CXCR4 expression via the ERK pathway in hepatocellular carcinoma. Oncoimmunology. 2018; 7:e1408745. 10.1080/2162402X.2017.1408745 29399411 PMC5790393

[103] Zhang X, Chen Y, Hao L, Hou A, Chen X, Li Y, Wang R, Luo P, Ruan Z, Ou J, Shi C, Miao H, Liang H. Macrophages induce resistance to 5-fluorouracil chemotherapy in colorectal cancer through the release of putrescine. Cancer Lett. 2016; 381:305–13. 10.1016/j.canlet.2016.08.004 27514455

[104] Wei C, Yang C, Wang S, Shi D, Zhang C, Lin X, Xiong B. M2 macrophages confer resistance to 5-fluorouracil in colorectal cancer through the activation of CCL22/PI3K/AKT signaling. Onco Targets Ther. 2019; 12:3051–63. 10.2147/OTT.S198126 31114248 PMC6489624

[105] Halbrook CJ, Pontious C, Kovalenko I, Lapienyte L, Dreyer S, Lee HJ, Thurston G, Zhang Y, Lazarus J, Sajjakulnukit P, Hong HS, Kremer DM, Nelson BS, et al. Macrophage-Released Pyrimidines Inhibit Gemcitabine Therapy in Pancreatic Cancer. Cell Metab. 2019; 29:1390–9.e6. 10.1016/j.cmet.2019.02.001 30827862 PMC6602533

[106] Spek CA, Aberson HL, Duitman J. Macrophage C/EBPδ Drives Gemcitabine, but Not 5-FU or Paclitaxel, Resistance of Pancreatic Cancer Cells in a Deoxycytidine-Dependent Manner. Biomedicines. 2022; 10:219. 10.3390/biomedicines10020219 35203429 PMC8869168

[107] Fu XT, Song K, Zhou J, Shi YH, Liu WR, Shi GM, Gao Q, Wang XY, Ding ZB, Fan J. Tumor-associated macrophages modulate resistance to oxaliplatin via inducing autophagy in hepatocellular carcinoma. Cancer Cell Int. 2019; 19:71. 10.1186/s12935-019-0771-8 30962765 PMC6434873

[108] Aslan K, Turco V, Blobner J, Sonner JK, Liuzzi AR, Núñez NG, De Feo D, Kickingereder P, Fischer M, Green E, Sadik A, Friedrich M, Sanghvi K, et al. Heterogeneity of response to immune checkpoint blockade in hypermutated experimental gliomas. Nat Commun. 2020; 11:931. 10.1038/s41467-020-14642-0 32071302 PMC7028933

[109] Lu JC, Zhang PF, Huang XY, Guo XJ, Gao C, Zeng HY, Zheng YM, Wang SW, Cai JB, Sun QM, Shi YH, Zhou J, Ke AW, et al. Amplification of spatially isolated adenosine pathway by tumor-macrophage interaction induces anti-PD1 resistance in hepatocellular carcinoma. J Hematol Oncol. 2021; 14:200. 10.1186/s13045-021-01207-x 34838121 PMC8627086

[110] House IG, Savas P, Lai J, Chen AXY, Oliver AJ, Teo ZL, Todd KL, Henderson MA, Giuffrida L, Petley EV, Sek K, Mardiana S, Gide TN, et al. Macrophage-Derived CXCL9 and CXCL10 Are Required for Antitumor Immune Responses Following Immune Checkpoint Blockade. Clin Cancer Res. 2020; 26:487–504. 10.1158/1078-0432.CCR-19-1868 31636098

[111] Ma S, Zhang L, Ren Y, Dai W, Chen T, Luo L, Zeng J, Mi K, Lang J, Cao B. Epiregulin confers EGFR-TKI resistance via EGFR/ErbB2 heterodimer in non-small cell lung cancer. Oncogene. 2021; 40:2596–609. 10.1038/s41388-021-01734-4 33750895

[112] Wang DH, Lee HS, Yoon D, Berry G, Wheeler TM, Sugarbaker DJ, Kheradmand F, Engleman E, Burt BM. Progression of EGFR-Mutant Lung Adenocarcinoma is Driven By Alveolar Macrophages. Clin Cancer Res. 2017; 23:778–88. 10.1158/1078-0432.CCR-15-2597 27496865

[113] Liu Y, Ji X, Kang N, Zhou J, Liang X, Li J, Han T, Zhao C, Yang T. Tumor necrosis factor α inhibition overcomes immunosuppressive M2b macrophage-induced bevacizumab resistance in triple-negative breast cancer. Cell Death Dis. 2020; 11:993. 10.1038/s41419-020-03161-x 33214550 PMC7678839

[114] Wei Q, Singh O, Ekinci C, Gill J, Li M, Mamatjan Y, Karimi S, Bunda S, Mansouri S, Aldape K, Zadeh G. TNFα secreted by glioma associated macrophages promotes endothelial activation and resistance against anti-angiogenic therapy. Acta Neuropathol Commun. 2021; 9:67. 10.1186/s40478-021-01163-0 33853689 PMC8048292

[115] Squadrito ML, De Palma M. Macrophage regulation of tumor angiogenesis: implications for cancer therapy. Mol Aspects Med. 2011; 32:123–45. 10.1016/j.mam.2011.04.005 21565215

[116] Deng L, Stafford JH, Liu SC, Chernikova SB, Merchant M, Recht L, Martin Brown J. SDF-1 Blockade Enhances Anti-VEGF Therapy of Glioblastoma and Can Be Monitored by MRI. Neoplasia. 2017; 19:1–7. 10.1016/j.neo.2016.11.010 27940247 PMC5149063

[117] Geissmann F, Manz MG, Jung S, Sieweke MH, Merad M, Ley K. Development of monocytes, macrophages, and dendritic cells. Science. 2010; 327:656–61. 10.1126/science.1178331 20133564 PMC2887389

[118] Ginhoux F, Schultze JL, Murray PJ, Ochando J, Biswas SK. New insights into the multidimensional concept of macrophage ontogeny, activation and function. Nat Immunol. 2016; 17:34–40. 10.1038/ni.3324 26681460

[119] Shih CT, Shiau CW, Chen YL, Chen LJ, Chao TI, Wang CY, Huang CY, Hung MH, Chen KF. TD-92, a novel erlotinib derivative, depletes tumor-associated macrophages in non-small cell lung cancer via down-regulation of CSF-1R and enhances the anti-tumor effects of anti-PD-1. Cancer Lett. 2021; 498:142–51. 10.1016/j.canlet.2020.10.043 33232786

[120] Lyons YA, Pradeep S, Wu SY, Haemmerle M, Hansen JM, Wagner MJ, Villar-Prados A, Nagaraja AS, Dood RL, Previs RA, Hu W, Zhao Y, Mak DH, et al. Macrophage depletion through colony stimulating factor 1 receptor pathway blockade overcomes adaptive resistance to anti-VEGF therapy. Oncotarget. 2017; 8:96496–505. 10.18632/oncotarget.20410 29228548 PMC5722500

[121] Brana I, Calles A, LoRusso PM, Yee LK, Puchalski TA, Seetharam S, Zhong B, de Boer CJ, Tabernero J, Calvo E. Carlumab, an anti-C-C chemokine ligand 2 monoclonal antibody, in combination with four chemotherapy regimens for the treatment of patients with solid tumors: an open-label, multicenter phase 1b study. Target Oncol. 2015; 10:111–23. 10.1007/s11523-014-0320-2 24928772

[122] Sandhu SK, Papadopoulos K, Fong PC, Patnaik A, Messiou C, Olmos D, Wang G, Tromp BJ, Puchalski TA, Balkwill F, Berns B, Seetharam S, de Bono JS, Tolcher AW. A first-in-human, first-in-class, phase I study of carlumab (CNTO 888), a human monoclonal antibody against CC-chemokine ligand 2 in patients with solid tumors. Cancer Chemother Pharmacol. 2013; 71:1041–50. 10.1007/s00280-013-2099-8 23385782

[123] Pienta KJ, Machiels JP, Schrijvers D, Alekseev B, Shkolnik M, Crabb SJ, Li S, Seetharam S, Puchalski TA, Takimoto C, Elsayed Y, Dawkins F, de Bono JS. Phase 2 study of carlumab (CNTO 888), a human monoclonal antibody against CC-chemokine ligand 2 (CCL2), in metastatic castration-resistant prostate cancer. Invest New Drugs. 2013; 31:760–8. 10.1007/s10637-012-9869-8 22907596

[124] Jung K, Heishi T, Incio J, Huang Y, Beech EY, Pinter M, Ho WW, Kawaguchi K, Rahbari NN, Chung E, Kim JK, Clark JW, Willett CG, et al. Targeting CXCR4-dependent immunosuppressive Ly6C(low) monocytes improves antiangiogenic therapy in colorectal cancer. Proc Natl Acad Sci U S A. 2017; 114:10455–60. 10.1073/pnas.1710754114 28900008 PMC5625928

[125] Schmid MC, Avraamides CJ, Dippold HC, Franco I, Foubert P, Ellies LG, Acevedo LM, Manglicmot JR, Song X, Wrasidlo W, Blair SL, Ginsberg MH, Cheresh DA, et al. Receptor tyrosine kinases and TLR/IL1Rs unexpectedly activate myeloid cell PI3kγ, a single convergent point promoting tumor inflammation and progression. Cancer Cell. 2011; 19:715–27. 10.1016/j.ccr.2011.04.016 21665146 PMC3144144

[126] Li J, Kaneda MM, Ma J, Li M, Shepard RM, Patel K, Koga T, Sarver A, Furnari F, Xu B, Dhawan S, Ning J, Zhu H, et al. PI3Kγ inhibition suppresses microglia/TAM accumulation in glioblastoma microenvironment to promote exceptional temozolomide response. Proc Natl Acad Sci U S A. 2021; 118:e2009290118. 10.1073/pnas.2009290118 33846242 PMC8072253

[127] Qin H, Yu H, Sheng J, Zhang D, Shen N, Liu L, Tang Z, Chen X. PI3Kgamma Inhibitor Attenuates Immunosuppressive Effect of Poly(l-Glutamic Acid)-Combretastatin A4 Conjugate in Metastatic Breast Cancer. Adv Sci (Weinh). 2019; 6:1900327. 10.1002/advs.201900327 31380170 PMC6662090

[128] Vidyarthi A, Khan N, Agnihotri T, Negi S, Das DK, Aqdas M, Chatterjee D, Colegio OR, Tewari MK, Agrewala JN. TLR-3 Stimulation Skews M2 Macrophages to M1 Through IFN-αβ Signaling and Restricts Tumor Progression. Front Immunol. 2018; 9:1650. 10.3389/fimmu.2018.01650 30072995 PMC6060442

[129] Anfray C, Mainini F, Digifico E, Maeda A, Sironi M, Erreni M, Anselmo A, Ummarino A, Gandoy S, Expósito F, Redrado M, Serrano D, Calvo A, et al. Intratumoral combination therapy with poly(I:C) and resiquimod synergistically triggers tumor-associated macrophages for effective systemic antitumoral immunity. J Immunother Cancer. 2021; 9:e002408. 10.1136/jitc-2021-002408 34531246 PMC8449972

[130] Liu Z, Xie Y, Xiong Y, Liu S, Qiu C, Zhu Z, Mao H, Yu M, Wang X. TLR 7/8 agonist reverses oxaliplatin resistance in colorectal cancer via directing the myeloid-derived suppressor cells to tumoricidal M1-macrophages. Cancer Lett. 2020; 469:173–85. 10.1016/j.canlet.2019.10.020 31629935

[131] Ni K, Luo T, Culbert A, Kaufmann M, Jiang X, Lin W. Nanoscale Metal-Organic Framework Co-delivers TLR-7 Agonists and Anti-CD47 Antibodies to Modulate Macrophages and Orchestrate Cancer Immunotherapy. J Am Chem Soc. 2020; 142:12579–84. 10.1021/jacs.0c05039 32658476

[132] Vonderheide RH, Glennie MJ. Agonistic CD40 antibodies and cancer therapy. Clin Cancer Res. 2013; 19:1035–43. 10.1158/1078-0432.CCR-12-2064 23460534 PMC3590838

[133] Beatty GL, Chiorean EG, Fishman MP, Saboury B, Teitelbaum UR, Sun W, Huhn RD, Song W, Li D, Sharp LL, Torigian DA, O'Dwyer PJ, Vonderheide RH. CD40 agonists alter tumor stroma and show efficacy against pancreatic carcinoma in mice and humans. Science. 2011; 331:1612–6. 10.1126/science.1198443 21436454 PMC3406187

[134] Vonderheide RH, Bajor DL, Winograd R, Evans RA, Bayne LJ, Beatty GL. CD40 immunotherapy for pancreatic cancer. Cancer Immunol Immunother. 2013; 62:949–54. 10.1007/s00262-013-1427-5 23589109 PMC3731141

[135] Zhang JQ, Zeng S, Vitiello GA, Seifert AM, Medina BD, Beckman MJ, Loo JK, Santamaria-Barria J, Maltbaek JH, Param NJ, Moral JA, Zhao JN, Balachandran V, et al. Macrophages and CD8^+^ T Cells Mediate the Antitumor Efficacy of Combined CD40 Ligation and Imatinib Therapy in Gastrointestinal Stromal Tumors. Cancer Immunol Res. 2018; 6:434–47. 10.1158/2326-6066.CIR-17-0345 29467128 PMC6203303

[136] Wang L, Wang D, Sonzogni O, Ke S, Wang Q, Thavamani A, Batalini F, Stopka SA, Regan MS, Vandal S, Tian S, Pinto J, Cyr AM, et al. PARP-inhibition reprograms macrophages toward an anti-tumor phenotype. Cell Rep. 2022; 41:111462. 10.1016/j.celrep.2022.111462 36223740 PMC9727835

[137] Liu Y, Xue R, Duan X, Shang X, Wang M, Wang F, Zhu L, Zhang L, Ge X, Zhao X, Guo H, Wang Z, Zhang L, et al. PARP inhibition synergizes with CD47 blockade to promote phagocytosis by tumor-associated macrophages in homologous recombination-proficient tumors. Life Sci. 2023; 326:121790. 10.1016/j.lfs.2023.121790 37211345

[138] Jin H, He Y, Zhao P, Hu Y, Tao J, Chen J, Huang Y. Targeting lipid metabolism to overcome EMT-associated drug resistance via integrin β3/FAK pathway and tumor-associated macrophage repolarization using legumain-activatable delivery. Theranostics. 2019; 9:265–78. 10.7150/thno.27246 30662566 PMC6332796

[139] Pozzi LA, Maciaszek JW, Rock KL. Both dendritic cells and macrophages can stimulate naive CD8 T cells in vivo to proliferate, develop effector function, and differentiate into memory cells. J Immunol. 2005; 175:2071–81. 10.4049/jimmunol.175.4.2071 16081773

[140] Wynn TA, Chawla A, Pollard JW. Macrophage biology in development, homeostasis and disease. Nature. 2013; 496:445–55. 10.1038/nature12034 23619691 PMC3725458

[141] Guerriero JL. Macrophages: The Road Less Traveled, Changing Anticancer Therapy. Trends Mol Med. 2018; 24:472–89. 10.1016/j.molmed.2018.03.006 29655673 PMC5927840

[142] Movahedi K, Laoui D, Gysemans C, Baeten M, Stangé G, Van den Bossche J, Mack M, Pipeleers D, In't Veld P, De Baetselier P, Van Ginderachter JA. Different tumor microenvironments contain functionally distinct subsets of macrophages derived from Ly6C(high) monocytes. Cancer Res. 2010; 70:5728–39. 10.1158/0008-5472.CAN-09-4672 20570887

[143] Doedens AL, Stockmann C, Rubinstein MP, Liao D, Zhang N, DeNardo DG, Coussens LM, Karin M, Goldrath AW, Johnson RS. Macrophage expression of hypoxia-inducible factor-1 alpha suppresses T-cell function and promotes tumor progression. Cancer Res. 2010; 70:7465–75. 10.1158/0008-5472.CAN-10-1439 20841473 PMC2948598

[144] Ruffell B, Chang-Strachan D, Chan V, Rosenbusch A, Ho CM, Pryer N, Daniel D, Hwang ES, Rugo HS, Coussens LM. Macrophage IL-10 blocks CD8+ T cell-dependent responses to chemotherapy by suppressing IL-12 expression in intratumoral dendritic cells. Cancer Cell. 2014; 26:623–37. 10.1016/j.ccell.2014.09.006 25446896 PMC4254570

[145] Matlack R, Yeh K, Rosini L, Gonzalez D, Taylor J, Silberman D, Pennello A, Riggs J. Peritoneal macrophages suppress T-cell activation by amino acid catabolism. Immunology. 2006; 117:386–95. 10.1111/j.1365-2567.2005.02312.x 16476058 PMC1782234

[146] Munn DH, Mellor AL. Macrophages and the regulation of self-reactive T cells. Curr Pharm Des. 2003; 9:257–64. 10.2174/1381612033392026 12570830

[147] Yao W, Ba Q, Li X, Li H, Zhang S, Yuan Y, Wang F, Duan X, Li J, Zhang W, Wang H. A Natural CCR2 Antagonist Relieves Tumor-associated Macrophage-mediated Immunosuppression to Produce a Therapeutic Effect for Liver Cancer. EBioMedicine. 2017; 22:58–67. 10.1016/j.ebiom.2017.07.014 28754304 PMC5552238

[148] Yin W, Li Y, Song Y, Zhang J, Wu C, Chen Y, Miao Y, Lin C, Lin Y, Yan D, Chen J, He R. CCRL2 promotes antitumor T-cell immunity via amplifying TLR4-mediated immunostimulatory macrophage activation. Proc Natl Acad Sci U S A. 2021; 118:e2024171118. 10.1073/pnas.2024171118 33846258 PMC8072249

[149] Fujiwara T, Yakoub MA, Chandler A, Christ AB, Yang G, Ouerfelli O, Rajasekhar VK, Yoshida A, Kondo H, Hata T, Tazawa H, Dogan Y, Moore MAS, et al. CSF1/CSF1R Signaling Inhibitor Pexidartinib (PLX3397) Reprograms Tumor-Associated Macrophages and Stimulates T-cell Infiltration in the Sarcoma Microenvironment. Mol Cancer Ther. 2021; 20:1388–99. 10.1158/1535-7163.MCT-20-0591 34088832 PMC9336538

[150] Song JS, Chang CC, Wu CH, Dinh TK, Jan JJ, Huang KW, Chou MC, Shiue TY, Yeh KC, Ke YY, Yeh TK, Ta YN, Lee CJ, et al. A highly selective and potent CXCR4 antagonist for hepatocellular carcinoma treatment. Proc Natl Acad Sci U S A. 2021; 118:e2015433118. 10.1073/pnas.2015433118 33753481 PMC8020795

[151] De Henau O, Rausch M, Winkler D, Campesato LF, Liu C, Cymerman DH, Budhu S, Ghosh A, Pink M, Tchaicha J, Douglas M, Tibbitts T, Sharma S, et al. Overcoming resistance to checkpoint blockade therapy by targeting PI3Kγ in myeloid cells. Nature. 2016; 539:443–7. 10.1038/nature20554 27828943 PMC5634331

[152] Quaranta V, Rainer C, Nielsen SR, Raymant ML, Ahmed MS, Engle DD, Taylor A, Murray T, Campbell F, Palmer DH, Tuveson DA, Mielgo A, Schmid MC. Macrophage-Derived Granulin Drives Resistance to Immune Checkpoint Inhibition in Metastatic Pancreatic Cancer. Cancer Res. 2018; 78:4253–69. 10.1158/0008-5472.CAN-17-3876 29789416 PMC6076440

[153] Kaneda MM, Messer KS, Ralainirina N, Li H, Leem CJ, Gorjestani S, Woo G, Nguyen AV, Figueiredo CC, Foubert P, Schmid MC, Pink M, Winkler DG, et al. PI3Kγ is a molecular switch that controls immune suppression. Nature. 2016; 539:437–42. 10.1038/nature19834 27642729 PMC5479689

[154] Peranzoni E, Lemoine J, Vimeux L, Feuillet V, Barrin S, Kantari-Mimoun C, Bercovici N, Guérin M, Biton J, Ouakrim H, Régnier F, Lupo A, Alifano M, et al. Macrophages impede CD8 T cells from reaching tumor cells and limit the efficacy of anti-PD-1 treatment. Proc Natl Acad Sci U S A. 2018; 115:E4041–50. 10.1073/pnas.1720948115 29632196 PMC5924916

[155] Barkal AA, Brewer RE, Markovic M, Kowarsky M, Barkal SA, Zaro BW, Krishnan V, Hatakeyama J, Dorigo O, Barkal LJ, Weissman IL. CD24 signalling through macrophage Siglec-10 is a target for cancer immunotherapy. Nature. 2019; 572:392–6. 10.1038/s41586-019-1456-0 31367043 PMC6697206

[156] Si Y, Zhang Y, Guan JS, Ngo HG, Totoro A, Singh AP, Chen K, Xu Y, Yang ES, Zhou L, Liu R, Liu XM. Anti-CD47 Monoclonal Antibody-Drug Conjugate: A Targeted Therapy to Treat Triple-Negative Breast Cancers. Vaccines (Basel). 2021; 9:882. 10.3390/vaccines9080882 34452008 PMC8402537

[157] Upton R, Banuelos A, Feng D, Biswas T, Kao K, McKenna K, Willingham S, Ho PY, Rosental B, Tal MC, Raveh T, Volkmer JP, Pegram MD, Weissman IL. Combining CD47 blockade with trastuzumab eliminates HER2-positive breast cancer cells and overcomes trastuzumab tolerance. Proc Natl Acad Sci U S A. 2021; 118:e2026849118. 10.1073/pnas.2026849118 34257155 PMC8307693

[158] Li L, Gong Y, Tang J, Yan C, Li L, Peng W, Cheng Z, Yu R, Xiang Q, Deng C, Mu J, Xia J, Luo X, et al. ZBTB28 inhibits breast cancer by activating IFNAR and dual blocking CD24 and CD47 to enhance macrophages phagocytosis. Cell Mol Life Sci. 2022; 79:83. 10.1007/s00018-021-04124-x 35048182 PMC11072821

[159] Osawa T, Tsuchida R, Muramatsu M, Yuasa Y, Shibuya M. Human glioblastoma cells exposed to long-term hypoxia and nutrient starvation stimulated induction of secondary T-cell leukemia in mice. Blood Cancer J. 2011; 1:e6. 10.1038/bcj.2011.5 22829112 PMC3255269

[160] Osawa T, Muramatsu M, Watanabe M, Shibuya M. Hypoxia and low-nutrition double stress induces aggressiveness in a murine model of melanoma. Cancer Sci. 2009; 100:844–51. 10.1111/j.1349-7006.2009.01105.x 19220297 PMC11159247

[161] Osawa T, Tsuchida R, Muramatsu M, Shimamura T, Wang F, Suehiro J, Kanki Y, Wada Y, Yuasa Y, Aburatani H, Miyano S, Minami T, Kodama T, Shibuya M. Inhibition of histone demethylase JMJD1A improves anti-angiogenic therapy and reduces tumor-associated macrophages. Cancer Res. 2013; 73:3019–28. 10.1158/0008-5472.CAN-12-3231 23492365

[162] Lu-Emerson C, Duda DG, Emblem KE, Taylor JW, Gerstner ER, Loeffler JS, Batchelor TT, Jain RK. Lessons from anti-vascular endothelial growth factor and anti-vascular endothelial growth factor receptor trials in patients with glioblastoma. J Clin Oncol. 2015; 33:1197–213. 10.1200/JCO.2014.55.9575 25713439 PMC4517055

[163] Batchelor TT, Mulholland P, Neyns B, Nabors LB, Campone M, Wick A, Mason W, Mikkelsen T, Phuphanich S, Ashby LS, Degroot J, Gattamaneni R, Cher L, et al. Phase III randomized trial comparing the efficacy of cediranib as monotherapy, and in combination with lomustine, versus lomustine alone in patients with recurrent glioblastoma. J Clin Oncol. 2013; 31:3212–8. 10.1200/JCO.2012.47.2464 23940216 PMC4021043

[164] Batchelor TT, Duda DG, di Tomaso E, Ancukiewicz M, Plotkin SR, Gerstner E, Eichler AF, Drappatz J, Hochberg FH, Benner T, Louis DN, Cohen KS, Chea H, et al. Phase II study of cediranib, an oral pan-vascular endothelial growth factor receptor tyrosine kinase inhibitor, in patients with recurrent glioblastoma. J Clin Oncol. 2010; 28:2817–23. 10.1200/JCO.2009.26.3988 20458050 PMC2903316

[165] Peterson TE, Kirkpatrick ND, Huang Y, Farrar CT, Marijt KA, Kloepper J, Datta M, Amoozgar Z, Seano G, Jung K, Kamoun WS, Vardam T, Snuderl M, et al. Dual inhibition of Ang-2 and VEGF receptors normalizes tumor vasculature and prolongs survival in glioblastoma by altering macrophages. Proc Natl Acad Sci U S A. 2016; 113:4470–5. 10.1073/pnas.1525349113 27044097 PMC4843449

[166] Kloepper J, Riedemann L, Amoozgar Z, Seano G, Susek K, Yu V, Dalvie N, Amelung RL, Datta M, Song JW, Askoxylakis V, Taylor JW, Lu-Emerson C, et al. Ang-2/VEGF bispecific antibody reprograms macrophages and resident microglia to anti-tumor phenotype and prolongs glioblastoma survival. Proc Natl Acad Sci U S A. 2016; 113:4476–81. 10.1073/pnas.1525360113 27044098 PMC4843473

[167] Tap WD, Gelderblom H, Palmerini E, Desai J, Bauer S, Blay JY, Alcindor T, Ganjoo K, Martín-Broto J, Ryan CW, Thomas DM, Peterfy C, Healey JH, et al. Pexidartinib versus placebo for advanced tenosynovial giant cell tumour (ENLIVEN): a randomised phase 3 trial. Lancet. 2019; 394:478–87. 10.1016/S0140-6736(19)30764-0 31229240 PMC6860022

[168] Manji GA, Van Tine BA, Lee SM, Raufi AG, Pellicciotta I, Hirbe AC, Pradhan J, Chen A, Rabadan R, Schwartz GK. A Phase I Study of the Combination of Pexidartinib and Sirolimus to Target Tumor-Associated Macrophages in Unresectable Sarcoma and Malignant Peripheral Nerve Sheath Tumors. Clin Cancer Res. 2021; 27:5519–27. 10.1158/1078-0432.CCR-21-1779 34321280 PMC8530953

[169] Tap WD, Wainberg ZA, Anthony SP, Ibrahim PN, Zhang C, Healey JH, Chmielowski B, Staddon AP, Cohn AL, Shapiro GI, Keedy VL, Singh AS, Puzanov I, et al. Structure-Guided Blockade of CSF1R Kinase in Tenosynovial Giant-Cell Tumor. N Engl J Med. 2015; 373:428–37. 10.1056/NEJMoa1411366 26222558

[170] Cassier PA, Italiano A, Gomez-Roca C, Le Tourneau C, Toulmonde M, D'Angelo SP, Weber K, Loirat D, Jacob W, Jegg AM, Michielin F, Christen R, Watson C, et al. Long-term clinical activity, safety and patient-reported quality of life for emactuzumab-treated patients with diffuse-type tenosynovial giant-cell tumour. Eur J Cancer. 2020; 141:162–70. 10.1016/j.ejca.2020.09.038 33161240

[171] Gomez-Roca C, Cassier P, Zamarin D, Machiels JP, Perez Gracia JL, Stephen Hodi F, Taus A, Martinez Garcia M, Boni V, Eder JP, Hafez N, Sullivan R, Mcdermott D, et al. Anti-CSF-1R emactuzumab in combination with anti-PD-L1 atezolizumab in advanced solid tumor patients naïve or experienced for immune checkpoint blockade. J Immunother Cancer. 2022; 10:e004076. 10.1136/jitc-2021-004076 35577503 PMC9114963

[172] Gomez-Roca CA, Italiano A, Le Tourneau C, Cassier PA, Toulmonde M, D'Angelo SP, Campone M, Weber KL, Loirat D, Cannarile MA, Jegg AM, Ries C, Christen R, et al. Phase I study of emactuzumab single agent or in combination with paclitaxel in patients with advanced/metastatic solid tumors reveals depletion of immunosuppressive M2-like macrophages. Ann Oncol. 2019; 30:1381–92. 10.1093/annonc/mdz163 31114846 PMC8887589

[173] Machiels JP, Gomez-Roca C, Michot JM, Zamarin D, Mitchell T, Catala G, Eberst L, Jacob W, Jegg AM, Cannarile MA, Watson C, Babitzki G, Korski K, et al. Phase Ib study of anti-CSF-1R antibody emactuzumab in combination with CD40 agonist selicrelumab in advanced solid tumor patients. J Immunother Cancer. 2020; 8:e001153. 10.1136/jitc-2020-001153 33097612 PMC7590375

[174] Xu J, Li J, Bai C, Xu N, Zhou Z, Li Z, Zhou C, Jia R, Lu M, Cheng Y, Mao C, Wang W, Cheng K, et al. Surufatinib in Advanced Well-Differentiated Neuroendocrine Tumors: A Multicenter, Single-Arm, Open-Label, Phase Ib/II Trial. Clin Cancer Res. 2019; 25:3486–94. 10.1158/1078-0432.CCR-18-2994 30833272

[175] Johnson M, Dudek AZ, Sukari A, Call J, Kunk PR, Lewis K, Gainor JF, Sarantopoulos J, Lee P, Golden A, Harney A, Rothenberg SM, Zhang Y, Goldman JW. ARRY-382 in Combination with Pembrolizumab in Patients with Advanced Solid Tumors: Results from a Phase 1b/2 Study. Clin Cancer Res. 2022; 28:2517–26. 10.1158/1078-0432.CCR-21-3009 35302585 PMC9359741

[176] Haag GM, Springfeld C, Grün B, Apostolidis L, Zschäbitz S, Dietrich M, Berger AK, Weber TF, Zoernig I, Schaaf M, Waberer L, Müller DW, Al-Batran SE, et al. Pembrolizumab and maraviroc in refractory mismatch repair proficient/microsatellite-stable metastatic colorectal cancer - The PICCASSO phase I trial. Eur J Cancer. 2022; 167:112–22. 10.1016/j.ejca.2022.03.017 35427833

[177] Irenaeus SMM, Nielsen D, Ellmark P, Yachnin J, Deronic A, Nilsson A, Norlén P, Veitonmäki N, Wennersten CS, Ullenhag GJ. First-in-human study with intratumoral administration of a CD40 agonistic antibody, ADC-1013, in advanced solid malignancies. Int J Cancer. 2019; 145:1189–99. 10.1002/ijc.32141 30664811

[178] Byrne KT, Betts CB, Mick R, Sivagnanam S, Bajor DL, Laheru DA, Chiorean EG, O'Hara MH, Liudahl SM, Newcomb C, Alanio C, Ferreira AP, Park BS, et al. Neoadjuvant Selicrelumab, an Agonist CD40 Antibody, Induces Changes in the Tumor Microenvironment in Patients with Resectable Pancreatic Cancer. Clin Cancer Res. 2021; 27:4574–86. 10.1158/1078-0432.CCR-21-1047 34112709 PMC8667686

[179] Padrón LJ, Maurer DM, O'Hara MH, O'Reilly EM, Wolff RA, Wainberg ZA, Ko AH, Fisher G, Rahma O, Lyman JP, Cabanski CR, Yu JX, Pfeiffer SM, et al. Sotigalimab and/or nivolumab with chemotherapy in first-line metastatic pancreatic cancer: clinical and immunologic analyses from the randomized phase 2 PRINCE trial. Nat Med. 2022; 28:1167–77. 10.1038/s41591-022-01829-9 35662283 PMC9205784

[180] Flinn IW, O'Brien S, Kahl B, Patel M, Oki Y, Foss FF, Porcu P, Jones J, Burger JA, Jain N, Kelly VM, Allen K, Douglas M, et al. Duvelisib, a novel oral dual inhibitor of PI3K-δ,γ, is clinically active in advanced hematologic malignancies. Blood. 2018; 131:877–87. 10.1182/blood-2017-05-786566 29191916 PMC6033052

[181] Shayan G, Kansy BA, Gibson SP, Srivastava RM, Bryan JK, Bauman JE, Ohr J, Kim S, Duvvuri U, Clump DA, Heron DE, Johnson JT, Hershberg RM, Ferris RL. Phase Ib Study of Immune Biomarker Modulation with Neoadjuvant Cetuximab and TLR8 Stimulation in Head and Neck Cancer to Overcome Suppressive Myeloid Signals. Clin Cancer Res. 2018; 24:62–72. 10.1158/1078-0432.CCR-17-0357 29061643 PMC5754237

[182] Kim MM, Parmar HA, Schipper M, Devasia T, Aryal MP, Kesari S, O'Day S, Morikawa A, Spratt DE, Junck L, Mammoser A, Hayman JA, Lawrence TS, et al. BRAINSTORM: A Multi-Institutional Phase 1/2 Study of RRx-001 in Combination With Whole Brain Radiation Therapy for Patients With Brain Metastases. Int J Radiat Oncol Biol Phys. 2020; 107:478–86. 10.1016/j.ijrobp.2020.02.639 32169409

[183] Lee MJ, Tomita Y, Yuno A, Lee S, Abrouk NE, Oronsky B, Caroen S, Trepel JB. Results from a biomarker study to accompany a phase II trial of RRx-001 with reintroduced platinum-based chemotherapy in relapsed small cell carcinoma. Expert Opin Investig Drugs. 2021; 30:177–83. 10.1080/13543784.2021.1863947 33306414 PMC9169989

[184] Reid TR, Abrouk N, Caroen S, Oronsky B, Stirn M, Larson C, Beale K, Knox SJ, Fisher G. ROCKET: Phase II Randomized, Active-controlled, Multicenter Trial to Assess the Safety and Efficacy of RRx-001 + Irinotecan vs. Single-agent Regorafenib in Third/Fourth Line Colorectal Cancer. Clin Colorectal Cancer. 2023; 22:92–9. 10.1016/j.clcc.2022.11.003 36529613

[185] Advani R, Flinn I, Popplewell L, Forero A, Bartlett NL, Ghosh N, Kline J, Roschewski M, LaCasce A, Collins GP, Tran T, Lynn J, Chen JY, et al. CD47 Blockade by Hu5F9-G4 and Rituximab in Non-Hodgkin's Lymphoma. N Engl J Med. 2018; 379:1711–21. 10.1056/NEJMoa1807315 30380386 PMC8058634

[186] Sikic BI, Lakhani N, Patnaik A, Shah SA, Chandana SR, Rasco D, Colevas AD, O'Rourke T, Narayanan S, Papadopoulos K, Fisher GA, Villalobos V, Prohaska SS, et al. First-in-Human, First-in-Class Phase I Trial of the Anti-CD47 Antibody Hu5F9-G4 in Patients With Advanced Cancers. J Clin Oncol. 2019; 37:946–53. 10.1200/JCO.18.02018 30811285 PMC7186585

[187] Ansell SM, Maris MB, Lesokhin AM, Chen RW, Flinn IW, Sawas A, Minden MD, Villa D, Percival MM, Advani AS, Foran JM, Horwitz SM, Mei MG, et al. Phase I Study of the CD47 Blocker TTI-621 in Patients with Relapsed or Refractory Hematologic Malignancies. Clin Cancer Res. 2021; 27:2190–9. 10.1158/1078-0432.CCR-20-3706 33451977

[188] Luke JJ, Barlesi F, Chung K, Tolcher AW, Kelly K, Hollebecque A, Le Tourneau C, Subbiah V, Tsai F, Kao S, Cassier PA, Khasraw M, Kindler HL, et al. Phase I study of ABBV-428, a mesothelin-CD40 bispecific, in patients with advanced solid tumors. J Immunother Cancer. 2021; 9:e002015. 10.1136/jitc-2020-002015 33608377 PMC7898862

[189] Weiss SA, Djureinovic D, Jessel S, Krykbaeva I, Zhang L, Jilaveanu L, Ralabate A, Johnson B, Levit NS, Anderson G, Zelterman D, Wei W, Mahajan A, et al. A Phase I Study of APX005M and Cabiralizumab with or without Nivolumab in Patients with Melanoma, Kidney Cancer, or Non-Small Cell Lung Cancer Resistant to Anti-PD-1/PD-L1. Clin Cancer Res. 2021; 27:4757–67. 10.1158/1078-0432.CCR-21-0903 34140403 PMC9236708

